# STAT1 signaling controls cholesterol metabolism in epithelial cells and RSV-induced syncytia formation

**DOI:** 10.1038/s44298-026-00173-w

**Published:** 2026-02-16

**Authors:** Ayse Agac, Martin Ludlow, Marie-Christin Knittler, Albert D.M.E. Osterhaus, Guus F. Rimmelzwaan, Robert Meineke

**Affiliations:** Research Center for Emerging Infections and Zoonoses, University of Veterinary Medicine Hannover, Hannover, Germany

**Keywords:** Diseases, Immunology, Microbiology

## Abstract

Respiratory syncytial virus (RSV) is a leading cause of respiratory tract infections, leading to significant morbidity, hospitalizations, and mortality among high-risk populations. Despite the recent advent of vaccines and monoclonal antibodies, options to treat RSV infections are limited. Detailed understanding at the molecular level of virus-host interactions associated with disease severity may aid development of novel intervention strategies. In this study, we examined the role of transcription factor STAT1 in regulating cholesterol metabolism during RSV infection of epithelial-like cells. We demonstrated that CRISPR/Cas9-mediated *STAT1* knock-out affected activation of the SREBP-SCAP cholesterol biosynthesis pathway, leading to intracellular cholesterol accumulation and increased RSV-induced syncytia formation. Pharmacological reduction of cholesterol levels blunted RSV-induced syncytia formation and affected the stability of the RSV fusion protein. These findings reveal a STAT1-dependent immune-metabolic pathway that constrains RSV dissemination through syncytia formation, which could be a novel target for intervention strategies.

## Introduction

Respiratory syncytial virus (RSV) is a leading cause of severe lower respiratory infections and hospitalization in infants, older adults, and immunocompromised individuals worldwide, manifesting as bronchiolitis and pneumonia^1–4^. In 2019, over 38 million cases of lower respiratory tract infection (LRTI) were reported in children under 5 years old and adults over 60, leading to more than four million hospitalizations and 150,000 RSV-related deaths^5,6^. Effective vaccines for older adults and pregnant women (Abrysvo, Arexvy), as well as highly neutralizing monoclonal antibodies for high-risk infants (Palivizumab, Nirsevimab, Clesrovimab), have become available in recent years^7–14^. However, RSV-specific antiviral therapies remain limited, underscoring the need for a better understanding of virus-host interactions.

Ciliated airway epithelial cells are the primary targets for RSV infection, viral replication, and spread^15,16^. Upon infection, RSV causes distinct cytopathic effects, including loss of cilia, cell sloughing, syncytia formation, and mucosal hypersecretion^17^.

To control virus dissemination and reduce cytopathic effects, innate immune responses are initiated during the early phase of infection. Type I and III interferons (IFNs), secreted by infected epithelial cells, represent the first response to infection, stimulating cells in an autocrine and paracrine manner, thereby triggering signaling cascades that initiate transcription of antiviral genes. Among these, Janus kinase (JAK)/signal transducer and activator of transcription (STAT) pathways are highly conserved regulators of immune responses and cellular homeostasis^18^. Although seven different STAT proteins have been identified, STAT1 is regarded as the central factor in immune responses during viral infections as it is activated upon binding of type I, II, and III IFNs^18,19^. Activation of STAT1 occurs through phosphorylation, resulting in its homo- or hetero (STAT2/3/4/5)-dimerization and nuclear translocation, where it binds to DNA sequences in regulatory gene regions, thereby initiating the transcription of antiviral IFN-stimulated genes (ISGs)^20^. Genetic STAT1 deficiency is associated with increased susceptibility to severe viral infections^21–23^.

Beyond its canonical role as a regulator of immune gene expression, STAT1 is involved in the regulation of lipid metabolic processes during viral infections^20^. As a key component of mammalian membranes, cholesterol is an important factor in virus entry, replication, and egress^24–28^. Cholesterol metabolism is highly regulated by ‘sterol regulatory element-binding proteins’ (SREBP), a family of endoplasmic reticulum (ER)-bound transcription factors, and its escort protein ‘SREBP cleavage-activating protein’ (SCAP), controlling the expression of genes involved in the biosynthesis and uptake of cholesterol, fatty acids, phospholipids, and triglycerides^29,30^. SREBP-SCAP activity is either directly regulated by STAT1 through transcriptional control of SREBP expression or indirectly via STAT1-mediated expression of cholesterol hydroxylases and, consequently, oxysterols, which act as negative regulators of the SREBP pathway by binding to ‘Insulin-induced Genes’ (INSIGs), transmembrane proteins responsible for retaining the SREBP-SCAP complex within the ER^31–34^. As a consequence of STAT1-mediated positive regulation of SREBP-SCAP activity and oxysterol synthesis, a reduction in cholesterol biosynthesis, cholesterol efflux, and the formation of cholesterol-rich lipid rafts, along with an increase in cholesterol esterification, can be observed upon viral infection^20,35,36^.

Recent studies have shown that RSV can alter cholesterol synthesis and its intracellular distribution by activating the SREBP-SCAP pathway^25^. RSV-mediated blockage of cholesterol transport to the ER results in the accumulation of cholesterol in lysosomes, providing a storage site for RSV Fusion (F) protein and viral replication^25^. As a result of low cholesterol levels in the ER, the activated SREBP-SCAP axis induces the expression of cholesterol synthesis through 3-hydroxy-3-methyl-glutaryl-coenzyme A reductase (HMGCR), the rate-limiting enzyme during cholesterol synthesis^37^.

While cholesterol is pivotal for efficient viral entry, its higher abundance may also result in better viral spread, as shown in the context of SARS-CoV-2 infections, where increased intracellular cholesterol levels and cholesterol-rich nanodomains enhanced virus-mediated syncytia formation, which was reversed by the use of 25-hydroxycholesterol (25-HC) and cholesterol-lowering drugs^34,38–40^. Although previous studies have investigated the role of STAT signaling during RSV infections in epithelial cells, little is known about the role of STAT1 and the SREBP-axis in syncytia formation, a hallmark of severe infections^41^. To understand how STAT1 activation and consequently reduced cholesterol levels affect RSV cell-to-cell spread, we generated a CRISPR-Cas9-mediated STAT1 knockout (KO) in HEp-2 cells. Our data suggest a pivotal role of STAT1 signaling and regulation of cholesterol metabolism by 25-HC in the formation of syncytia in epithelial cells. We therefore present a model for understanding RSV-mediated syncytia formation in epithelial cells in the context of severe infections in infants, older adults, and immunocompromised individuals.

## Results

### Biallelic Knockout of STAT1 alters cellular cholesterol metabolism and homeostasis

**Fig. 1:**
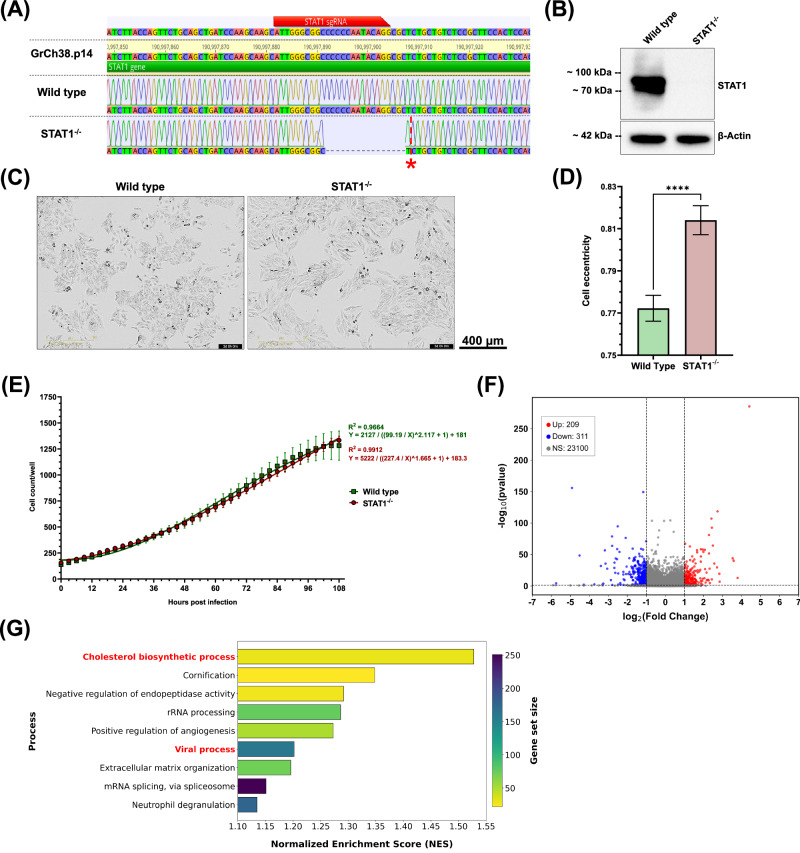
Confirmation of STAT1 deletion and characterization of STAT1^−/−^ HEp-2 cells. **A** Sequence alignment of wild-type and STAT1^−/−^ to STAT1 gene of the human genome (GrCh38.p14). The red bar indicates the binding site of sgRNAs. The red star indicates the 17 bp deletion on the second allele. **B** Western blot of whole cell lysates of wild-type and STAT1^−/−^ HEp-2 cells targeting STAT1 (top) and β-Actin (bottom) as loading control. **C** Bright-field IncuCyte live-cell images of wild-type (left) and STAT1^−/−^ HEp-2 (right) at 72 hours (h) post-seeding. Images representative of three independent experiments (10 technical replicates). Scale bar indicated next to images. **D** Quantification of cell eccentricity for wild-type and STAT1^−/−^ HEp-2 cells at 72 h post-seeding based on live cell images. Eccentricity was assessed at approximately 60% confluence, which was reached at 72 h post-seeding for both cell types. Minimum area, minimum eccentricity, and Hole fill were set to 600 μm^2^, 0.3, and 1000 μm^2^, respectively. Mean ± SD from three independent experiments, with 10 replicates each, are shown. Statistical analysis: Unpaired t-test. **E** Quantitative analysis of IncuCyte live-cell images for cells per well, with images taken every 3 h for 4.5 days. Mean ± SD of three independent experiments (10 technical replicates) is shown, with non-linear regressions applied for wild-type (green) and STAT1^−/−^ (red) HEp-2 cells. Statistical analysis: Mann-Whitney test for comparison of ranks. **F** Volcano plot demonstrating the number of differentially expressed genes between wild-type and STAT1^−/−^ HEp-2 cells with thresholds p < 0.05 and |Log2 Fold change | ≥ 1. **G** Bar plot of Gene Set Enrichment Analysis of Gene Ontology. The plot shows significantly enriched biological processes in STAT1^−/−^ compared to wild-type HEp-2 cells.

For the generation of a STAT1^−/−^ HEp-2 cell line, a CRISPR-Cas9-mediated knockout was performed targeting the first exon of the *STAT1* gene (ENSG00000115415). Oxford Nanopore sequencing of the resulting clone revealed a biallelic, compound heterozygous deletion of 16 base pairs (bp) and 17 bp, occurring just after the sgRNA binding site (Fig. 1A). We verified the knockout at protein level in the HEp-2 clone by western blotting for STAT1 (Fig. 1B). While STAT1 was abundant in wild-type HEp-2 cells, no expression was detected in the STAT1^−/−^ clone, confirming the knockout on both genetic and protein levels. Transcriptomic comparison of wild-type and STAT1^−/−^ HEp-2 cells further showed an almost two-fold reduction of STAT1 expression in STAT1^−/−^ cells, while a minor decrease in the expression of STAT1 dimerization partners (STAT2-5) was also observed (Supplementary Figure 1a). Since STAT1 is involved not only in immune response signaling but also in cellular homeostasis^18^, we continued to characterize the STAT1^−/−^ HEp-2 cell line in terms of cellular growth and morphology compared to wild-type HEp-2 cells. Cellular expansion of both wild-type and STAT1^−/−^ HEp-2 cells was quantified over 4.5 days using IncuCyte Live-cell imaging (Fig. 1C,D). Bright-field images of wild-type HEp-2 cells revealed a triangular to polygonal shape typical of epithelial cells (Fig. 1C, left), while STAT1^−/−^ HEp-2 cells were elongated and spindle-shaped (Fig. 1C, right). This observation was supported by the significantly increased eccentricity of STAT1^−/−^ cells compared to wild-type cells (Fig. 1D, Supplementary Figure 1b). Comparison of cell growth kinetics revealed no significant differences between wild-type and STAT1^−/−^ HEp-2 cells (*p* = 0.9572) (Fig. 1E). We continued to analyze the effect of STAT1 deletion in HEp-2 cells by RNA-Seq (Fig. 1E,F). Our analysis revealed that 209 genes were upregulated, and 311 genes were downregulated in STAT1^−/−^ HEp-2 cells compared to wild-type cells (Fig. 1F). Transcriptomic analysis of wild-type and STAT1^−/−^ HEp-2 cells was continued by Gene Set Enrichment Analysis (GSEA) using Gene ontologies (GO) terms, characterizing broad biological processes and functions, and Reactome databases, allowing mechanistically detailed comparison of signaling pathways. The GSEA GO analysis revealed significant enrichment of gene sets related to cholesterol biosynthetic processes, with a Normalized Enrichment Score (NES) of 1.53 (Fig. 1G). These findings are supported by Reactome pathway analysis, which highlights the enrichment of cholesterol biosynthesis-related pathways with NESs ranging from 1.35 to 1.5 (Supplementary Fig. 1b). Genes assigned to these processes can be classified into four clusters: 1) Genes directly involved in cholesterol biosynthesis (*HMGCS1, HMGCR, MVK, MVD, FDPS, FDFT1, SQLE, LSS, MSMO1, NSDHL, EBP, CYP51A1, TM7SF2, HSD17B7, DHCR7, DHCR24, SC5D, GGPS1*), 2) Transcription (co-)factors controlling cholesterol biosynthesis (*SREBF2*, *CREBBP*, *CARM1*, *TBL1X*, *NFYB*, *NFYC*, *NCOA6, MTF1*), 3) Indirect modulators of cholesterol homeostasis (*ACAA2*, *ACACA*, *SCD*, *ELOVL6*, *FASN*, *GPAM*), and 4) Regulators of cholesterol biosynthesis and homeostasis (*INSIG1*, *INSIG2*, *PRKAA2*, *ACAT2*, *KPNB1*, *SEC24C*). A summary of the clusters, the involved genes, and expression trend is provided in Supplemental Table 2.

Notably, expression of these genes was significantly lower in STAT1^−/−^ HEp-2 cells compared to expression in wild-type HEp-2 cells, except for *DHCR7*, *NFYB*, *SC5D*, and *INSIG2*, which were significantly higher in STAT1^−/−^ cells.

### No differences in RSV replication kinetics but increased cell fusion in STAT1^−/−^ HEp-2 cells

**Fig. 2:**
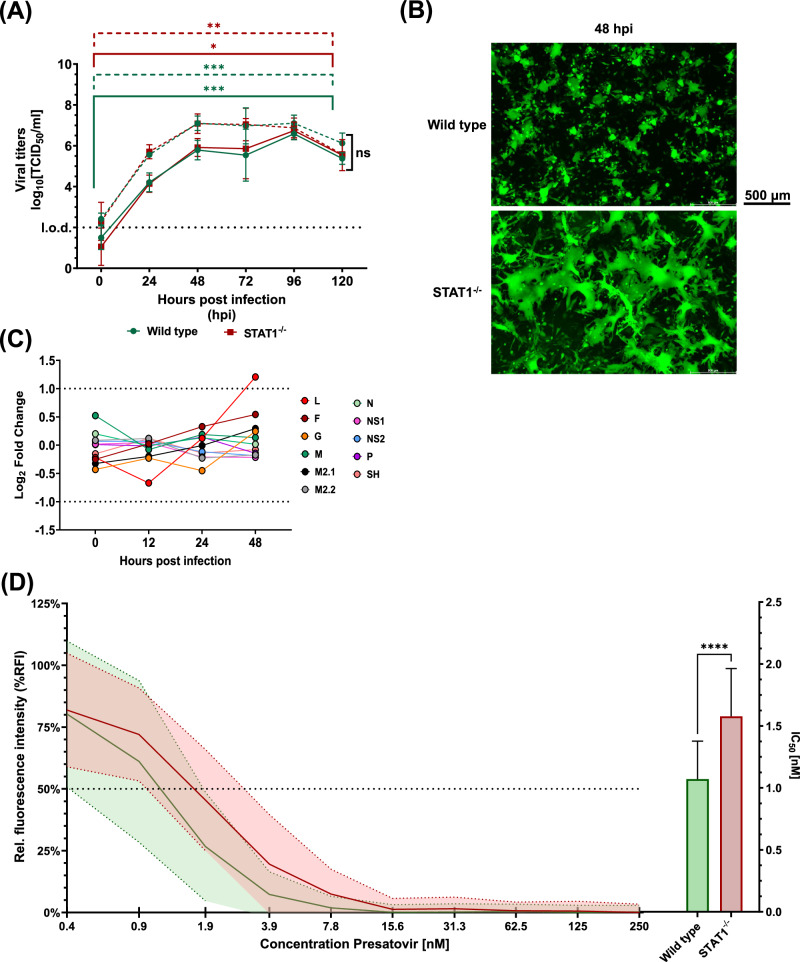
Characterization of RSV infection in wild-type and STAT1^−/−^ HEp-2 cells. **A** Viral replication kinetics of RSV-A-0594 (dashed lines) and rRSV-A-0594-eGFP (solid lines)-infected wild-type (green) and STAT1^−/−^ (red) HEp-2 cells (MOI 0.05). Mean ± SD of three independent experiments (four technical replicates) is shown. Statistical analysis: Two-way ANOVA with Fisher’s LSD test. **B** Fluorescence images of rRSV-A-0594-eGFP-infected (MOI 0.05) wild-type (top) and STAT1^−/−^ (bottom) Hep-2 cells at 48 hours post-infection (hpi). Images representative of three independent experiments (two technical replicates). Scale bar indicated next to images. **C** Graph showing the comparison of viral transcript numbers from RNA-Seq analysis between wild-type and STAT1^−/−^ HEp-2 cells from 0 to 72 hpi with thresholds set at p < 0.05 and |Log2 Fold change | ≥ 1. **D** Presatovir-based fusion inhibition assay on wild-type (green) and STAT1^−/−^ (red) HEp-2 cells at 72 hpi with rRSV-A-0594-eGFP (MOI 0.05). Fluorescence intensity (eGFP) relative to untreated, infected cells is shown. Solid lines indicate mean values, while dashed lines above and below each curve indicate the corresponding standard deviation (Mean ± SD). Horizontal dashed line and the right graph indicate Half Maximal Inhibitory Concentration (IC_50_) of wild-type (green) and STAT1^−/−^ (red) HEp-2 based on non-linear regression fitted to graphs. Mean ± SD of three independent experiments (eight technical replicates) is shown. Statistical analysis for IC_50_ comparison: Unpaired t-test with Welsh’s correction.

We continued to characterize the effect of STAT deletion in the context of viral infection. Since STAT1 signaling is pivotal for the initiation of antiviral responses, its absence may benefit viral replication and spread in STAT1^−/−^ HEp-2 cells. We quantified infectious viral particles in whole-cell preparations of RSV-A-0594 or rRSV-A-0594-eGFP-infected wild-type and STAT1^−/−^ HEp-2 cells for up to five days post-infection by TCID_50_ assay (according to Reed and Muench^42^). For both cell types, a significant increase in RSV titers was observed for both virus strains, with titers peaking at 96 hpi (Fig. 2A). Direct comparison of viral titers revealed no significant difference at any time point between the two cell types. Although viral titers did not differ significantly between wild-type and STAT1^−/−^ HEp-2 cells, we observed differential pathophysiology upon infection (Fig. 2B). Microscopic analysis of rRSV-A-0594-eGFP-infected wild-type and STAT1^−/−^ HEp-2 cells at 48 hpi revealed large syncytia formation in STAT1^−/−^ HEp-2 cells, which was observed for the wild-type HEp-2 cells only to a limited extent. RNA-Seq analysis of viral mRNA upon infection with rRSV-A-0594-eGFP revealed no significant differences in the abundance of viral transcripts at 0, 12, and 24 hpi (Fig. 2C, Supplementary Figure 2a), while at 48 hpi, a significantly increased expression was only observed for the viral polymerase (RSV L protein) in STAT1^−/−^ HEp-2 cells (Log_2_FC = 1.2). To characterize the effect of altered STAT1 signaling and increased syncytia formation on inhibition of RSV infection, fusion inhibition assays were performed using Presatovir, a small-molecule F protein inhibitor currently in clinical trials for therapeutic treatment of RSV-infected individuals (NCT04938830)^43–45^. A similar kinetic was observed between the two cell types and comparison of inhibition dynamics based on a non-linear fit of the data revealed no significant difference between wild-type and STAT1^−/−^ HEp-2 cells (P[Hill slope] = 0.1137) and in neither cell type eGFP expression was detectable at a Presatovir concentration of 125 nM (Fig. 2D, Supplementary Fig. 2b). Calculation of the half maximal inhibitory concentration (IC_50_) based on the non-linear fitted graphs showed a significantly increased value for STAT1^−/−^ HEp-2 (1.6 nM) compared to wild-type HEp-2 (1.1 nM).

### Cellular cholesterol levels affect fusion events upon RSV infection

**Fig. 3:**
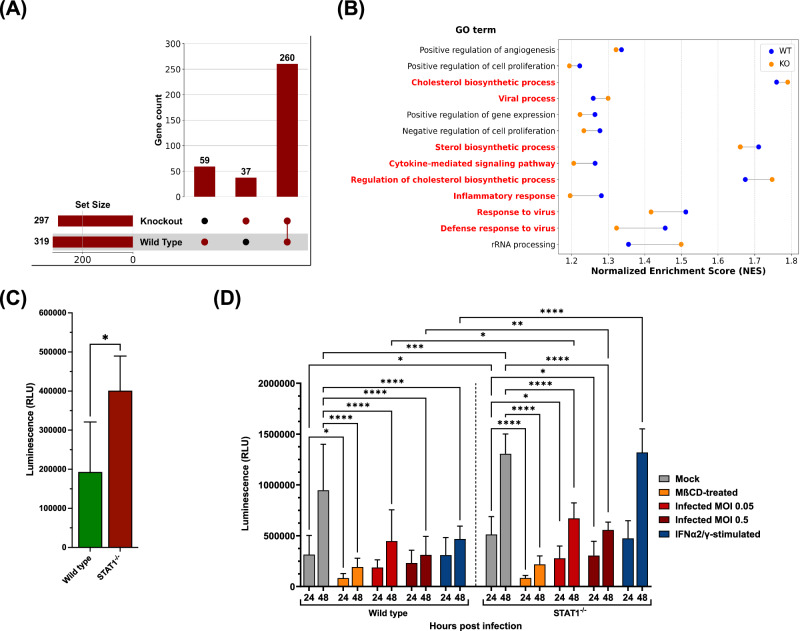
Baseline differences and quantification of free cholesterol in wild-type and STAT1^−/−^ HEp-2 cells. **A** UpSet plot demonstrating differentially expressed genes that are shared and unique to infected wild-type and STAT1^−/−^ HEp-2 after comparison to respective uninfected controls with thresholds *p* < 0.05 and |Log2 Fold change | ≥ 1. **B** Cleveland Dot Plot of Gene Set Enrichment Analysis (GSEA) of Gene Ontology comparing processes enriched in both infected wild-type and STAT1^−/−^ HEp-2 cells based on Normalized Enrichment Scores (NES). **C** Quantification of total free cholesterol by luminescent enzymatic assay in uninfected wild-type (green) and STAT1^−/−^ (red) HEp-2 cells. Statistical analysis: Mann-Whitney test. **D** Quantification of total free cholesterol by luminescent enzymatic assay in wild-type and STAT1^−/−^ HEp-2 cells, which were uninfected (grey), treated with 5 mM methyl-beta-cyclodextrin (MβCD, orange), infected at MOI 0.05 (light red), infected at MOI (dark red), or treated with 100 ng/ml IFN-α2 and 50 ng/mL IFN-γ at 24 and 48 hpi. Only significant differences between untreated and treated cells within a cell type or within treatments between cell types are shown. Statistical analysis: two-way ANOVA with Fisher’s LSD test. Mean ± SD of four independent experiments (two technical replicates) shown for (**C**, **D**).

We continued to analyze differences in gene expression upon rRSV-A-0594-eGFP infection of STAT1^−/−^ and wild-type HEp-2 cells using RNA-Seq. To exclude any differences in background gene expression between the two cell types, gene expression of infected STAT1^−/−^ and wild-type HEp-2 cells was first compared to the respective uninfected samples, followed by comparison of DEGs and GSEA of GO and Reactome databases between STAT1^−/−^ and wild-type HEp-2 cells. This comparison of DEGs showed that, after RSV infection, 59 genes were unique to wild-type HEp-2 cells, 37 genes were exclusively differentially expressed in STAT1^−/−^ HEp-2 cells, and 260 genes were differentially expressed in both cell types (Fig. 3A). We continued to analyze possible differences in DEGs, that were shared between the two cell types, by GSEA of GO (Fig. 3B) and Reactome pathway analysis (Supplementary Fig. 2c), as the majority of genes that were unique to infected wild-type HEp-2 cells were related to cytokine signaling and those unique to infected STAT1^−/−^ cells related to intracellular stress responses. Pathways and processes linked to inflammatory responses and interferon signaling in response to viral infections were enriched in both wild-type HEp-2 cells and STAT1^−/−^ cells, with higher NESs in wild-type HEp-2 cells. We categorized the genes assigned to these processes and pathways into six clusters: 1) ‘Innate immune sensors’ (e.g., *IFIH1* [Melanoma Differentiation-Associated protein 5, MDA-5], *DDX58* [Retinoic Acid-Inducible Gene I, RIG-I], *DHX58* [Laboratory of Genetics and Physiology 2, LGP2], *TMEM173* [Stimulator of Interferon Genes, STING], *IFI16*), 2) ‘Antigen processing and presentation’ (e.g., *HLA-B*, *TAP1*, *PSMB8*, *B2M*, *HLA-C*), 3) ‘Host factors involved in viral replication’ (e.g., *EIF4G1*, *NUP98*, *DDX3X*, *NXF1*, *RANBP2*), 4) ‘Stress response regulators’ (e.g., *BAX*, *BNIP3*, *CFLAR*, *HSPA8*, *CASP1* [caspase 1]), 5) ‘Immune signaling and transcription regulators’ (e.g., *STAT1*, *STAT3*, *JAK1*, *IRF7*, *IRAK1*), and 6) ‘Interferon-stimulated genes’ (e.g., *ISG15*, *IFIT1*, *RSAD2* [Viperin], *TRIM25*, *OAS1*). An overview of genes assigned to these clusters and their respective expression trend is provided in Supplemental Table 3. Most of the genes in clusters 1, 2, 4, 5, and 6 were significantly upregulated in infected wild-type HEp-2 and STAT1^−/−^ HEp-2 cells compared to the respective uninfected control, although gene expression levels were mainly lower in infected STAT1^−/−^ cells (especially for clusters 5 and 6). While the expression trends for clusters 1, 2, 4, 5, and 6 were comparable between infected wild-type and STAT1^−/−^ cells, we noticed differences in the expression of genes assigned to cluster 3 (‘Host factors involved in viral replication’). Most Cluster 3 genes were significantly downregulated in infected wild-type HEp-2 cells. In contrast, their expression was significantly upregulated in infected STAT1^−/−^ cells compared to the respective uninfected controls. Besides innate responses and interferon signaling, cholesterol biosynthesis-related processes and pathways were highly enriched in both cell types, with a stronger enrichment in STAT1^−/−^ cells compared to wild-type HEp-2 cells, except for the ‘sterol biosynthetic process’, which had a higher NES in wild-type HEp-2. Genes assigned to these processes were again clustered into four categories, based on their role during cholesterol biosynthesis, namely direct involvement (*FDPS*, *MSMO1*, *DHCR7*, *SQLE*, *MVD*, *HMGCR*, *HSD17B7*, *FDFT1*, *HMGCS1*, *LSS*, *DHCR24*, *EBP*, *MVK*, *CYP51A1*, *NSDHL*, *TM7SF2*, *GGP51*, *SC5D*, *IDI1*), transcription (co-)factors (*CARM1*, *SCAP*, *HELZ2*, *MTF1*, *CREBBP*, *TBL1X*, *NCOA6*, *SREBF2*), indirect modulators (*ACLY*, *CYB5R3*, *FASN*, *ELOVL6*, *ACACA*, *SCD*, *G6PD*), and direct regulators (*INSIG1/2*, *ACAT2*). The summary of the mentioned clusters, including assigned genes and their expression trend compared to uninfected samples, is provided in Supplemental Table 4. Notably, most of the mentioned genes were significantly downregulated in infected wild-type HEp-2 cells compared to uninfected cells, while the majority were significantly upregulated in STAT1^−/−^ HEp-2 cells. Similar to wild-type HEp-2 cells, expression of *FDFT1*, *DHCR24*, *TM7SF2*, *SC5D*, *IDI1* (all directly involved in cholesterol biosynthesis), and SCD (direct regulator) was significantly downregulated in STAT1^−/−^ HEp-2 cells compared to uninfected cells. Comparing transcriptomic data from wild-type and STAT1^−/−^ HEp-2 cells under both homeostatic and infection conditions revealed a link between STAT1 signaling and intracellular cholesterol metabolism. We sought to validate this finding with follow-up experiments focusing on the effect of absent STAT1 signaling on intracellular cholesterol levels. Lysed STAT1^−/−^ HEp-2 cells displayed a significant two-fold increase in total, free cholesterol levels compared to lysed, wild-type HEp-2 cells, in the absence of both infection and stimulation (Fig. 3c). We continued to assess the changes in cholesterol content upon infection, stimulation with IFN-α2/IFN-γ, or treatment with M${\rm{\beta }}$CD, a compound that depletes cholesterol from cellular membranes, at 24 and 48 h post-treatment (Fig. 3d). Treatment of wild-type cells with M${\rm{\beta }}$CD resulted in a significant decrease in free cholesterol compared to untreated cells, with over threefold and fivefold reductions observed at 24 and 48 hpi, respectively. Comparably, infection of wild-type HEp-2 with RSV-A-0594 (MOI 0.05 and MOI 0.5) and stimulation of cells with IFN-α2/IFN-γ resulted in significant decreases in free cholesterol levels by at least two-fold. Comparable to wild-type HEp-2, treatment of STAT1^−/−^ HEp-2 cells with MβCD resulted in a significant sixfold reduction in free cholesterol levels. Upon infection with RSV-A-0594 (MOI 0.05 and MOI 0.5), free cholesterol levels in STAT1^−/−^ HEp-2 were significantly decreased by at least 1.5-fold. In contrast to wild-type HEp-2 cells, stimulation of STAT1^−/−^ HEp-2 cells with IFN-α2/IFN-γ resulted in no significant changes at 24 (*p* = 0.6777) or 48 hpi (*p* = 0.8827) compared to uninfected, untreated STAT1^−/−^ HEp-2 cells. Significant differences between wild-type and STAT1^−/−^ HEp-2 cells were also observed. More specifically, free cholesterol levels were significantly elevated in untreated STAT1^−/−^ cells at 24 and 48 hpi (1.5-fold and 1.4-fold, respectively), at 48 hpi in infected samples (1.5-fold for MOI 0.05, 1.8-fold for MOI 0.5), as well as 48 h after stimulation with IFN-α2/IFN-γ (2.8-fold).

### Depletion of cholesterol in STAT1^−/−^ cells reduces syncytia size

**Fig. 4:**
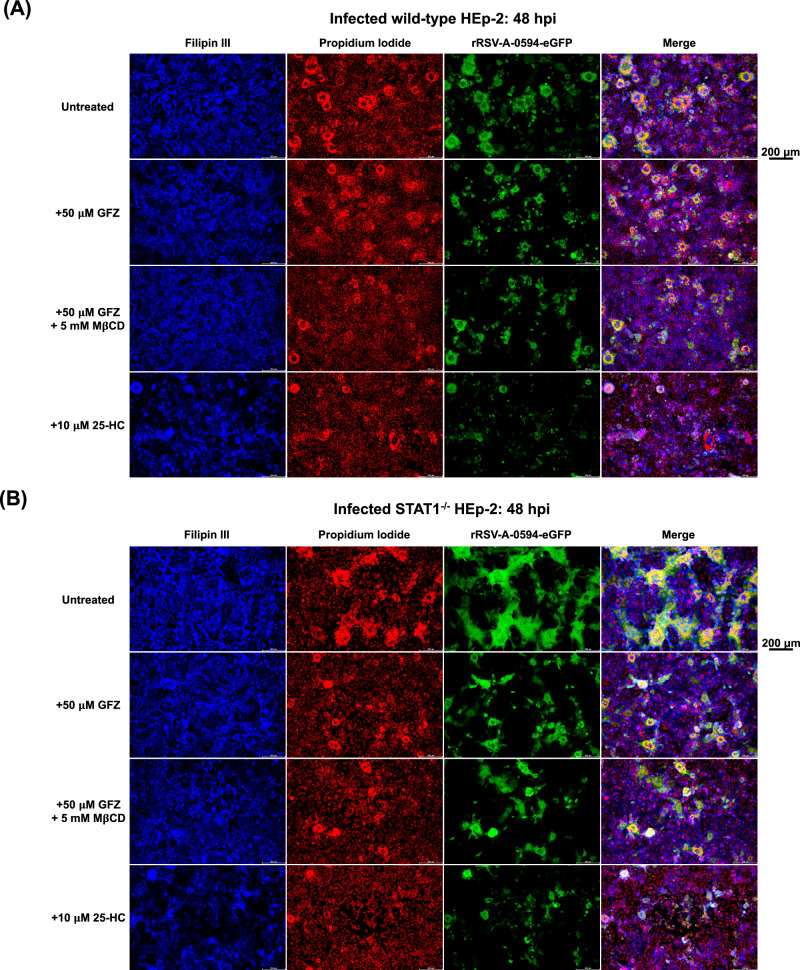
Dependence of syncytia formation on surface cholesterol levels. Representative fluorescence images of rRSV-A-0594-eGFP-infected wild-type (**A**) and STAT1^−/−^ (**B**) HEp-2 cells (MOI 0.05) at 48 hpi. Cells were left untreated (first row), treated with 50 μM Gemfibrozil (GFZ, second row), treated with 5 μM methyl-β-cyclodextrin (MβCD) and 50 μM GFZ (third row), or with 10 μM 25-hydroxycholesterol (25-HC, bottom panel). Free cholesterol in plasma membranes was visualized by Filipin III staining (0.05 mg/ml), and DNA was visualized by Propidium iodide (PI) staining (5 μg/ml). Images representative of three independent experiments (two technical replicates). Scale bar indicated next to images.

Since the data shown above reveal a link between functional STAT1 signaling and intracellular cholesterol levels, we further investigated the dependency of virus-mediated cell fusion on cellular cholesterol levels using fluorescence microscopy. We used Filipin III, a fluorescent cholesterol-specific probe, to visualize free cholesterol in the plasma membrane of rRSV-A-0594-eGFP-infected or uninfected wild-type and STAT1^−/−^ HEp-2 cells at 0 and 48 hpi (Fig. 4, Supplementary Figs. 3–5). To assess differences in signal intensity between cell types and treatments, Filipin III staining intensity was quantified, and corrected total cell fluorescence was calculated (Supplementary Figure 6). As observed in previous experiments, infection of STAT1^−/−^ cells induced increased syncytia formation compared to wild-type HEp-2 cells, while no significant differences in surface cholesterol levels were observed (Fig. 4, Supplementary Fig. 6). Next, we assessed the effect of reduced cholesterol levels on syncytia formation in infected cells by the addition of cholesterol-lowering agents. Treatment with GFZ, a PPAR-α agonist that primarily modulates lipid metabolism but also alters cholesterol transport, did not result in significant changes in Filipin III staining (Supplementary Figure 6), indicating that GFZ does not alter free cholesterol levels in the membrane but rather affects intracellular cholesterol levels. Depletion of cholesterol by MβCD treatment of wild-type and STAT1^−/−^ HEp-2 cells led to a significant reduction in surface free cholesterol at 0 hpi in both uninfected and infected cells (Supplementary Figs. 3, 4a, 5a, 6a,c). However, surface cholesterol levels returned to levels of untreated cells by 48 hpi, indicating that MβCD treatment only transiently affects cellular cholesterol levels (Fig. 4, Supplementary Figs. 4b, 5b, 6b,d). Although treatment of wild-type and STAT1^−/−^ HEp-2 cells with GFZ (with or without MβCD) did not alter surface cholesterol levels at 48 hpi, we observed a visible reduction in syncytia size in both cell types (Fig. 4). Incubation of wild-type and STAT1^−/−^ HEp-2 cells with 25-HC, an oxysterol and inhibitor of cholesterol biosynthesis by suppressing the activation of the SREBP pathway, resulted in a significant decrease in Filipin III staining at 48 hpi and, therefore, free cholesterol in the plasma membrane, in both infected (Fig. 4) and uninfected cells (Supplementary Figs. 4b, 5b, 6b,d), along with a visible reduction in syncytia size. While in GFZ/GFZ + MβCD-treated cells, syncytia still seemed to be slightly increased in STAT1^−/−^ cells compared to treated wild-type cells, no visible difference was noted between wild-type and STAT1^−/−^ HEp-2 cells upon 25-HC treatment with infection limited to individual cells. The corresponding bright-field images of infected/uninfected and treated/untreated wild-type and STAT1^−/−^ HEp-2 cells are shown in Supplementary Figs. 7 and 8.

**Fig. 5:**
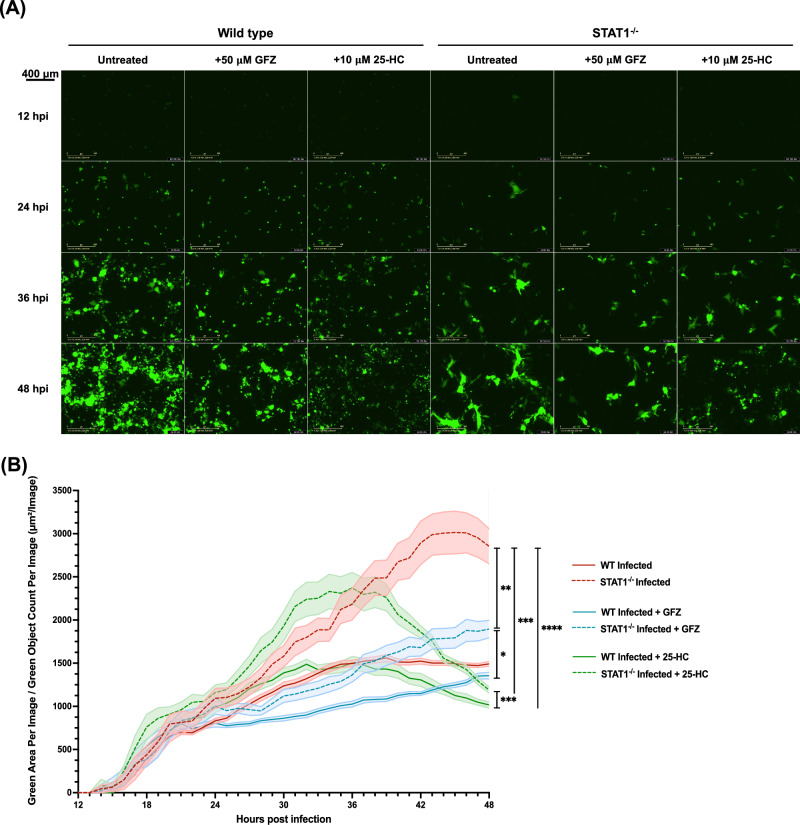
Dependence of syncytia formation on cellular cholesterol levels. **A** Fluorescence images from IncuCyte live-cell imaging of rRSV-A-0594-eGFP-infected wild-type (left) and STAT1^−/−^ (right) HEp-2 cells (MOI 0.05), which were left untreated (left column), treated with 50 μM Gemfibrozil (GFZ, middle column), or with 10 μM 25-hydroxycholesterol (25-HC, right column) at 12, 24, 36, and 48 hpi. Images representative of three independent (two technical replicates) experiments. Scale bar indicated next to images. **B** Quantification of syncytia size by IncuCyte live-cell imaging for green (fluorescent) area per image relative to green object count per image of rRSV-A-0594-eGFP-infected wild-type (solid lines) and STAT1^−/−^ (dashed lines) HEp-2 cells (MOI 0.05), either untreated (red), treated with 50 μM GFZ (blue), or 10 μM 25-HC (green). Images of cells were taken every hour for up to 48 hpi. Mean ± SEM of eight independent experiments (two technical replicates) is shown. Statistical analysis was conducted on the overall distribution of syncytia size during the course of infection using the Friedman test with Dunn’s multiple-comparison test.

We further validated a possible link between cholesterol levels and syncytia formation by quantifying differences in syncytia size in the absence or presence of cholesterol-lowering agents. To this end, rRSV-A-0594-eGFP-infected wild-type and STAT1^−/−^ HEp-2 cells were imaged over 48 h using an IncuCyte live-cell imager (Fig. 5a, Supplementary Figs. 9–12). Quantification of syncytia size relative to the number of eGFP-positive cells (Green Area per Image/ Green object count per image) and statistical comparison of syncytia size over the course of infection confirmed our previous observations (Fig. 5b). The size of eGFP+ cells was significantly smaller in wild-type HEp-2 cells compared to STAT1^−/−^ HEp-2 cells (red lines). Syncytia size in wild-type HEp-2 cells reached a plateau at 36 hpi, while it peaked at 45 hpi for STAT1^−/−^ cells. No significant changes were observed in syncytia size upon treatment of infected wild-type HEp-2 cells with cholesterol-lowering drugs (*p* > 0.99), whereas treatment of infected STAT1^−/−^ cells resulted in a significant reduction of syncytia size (1.3-fold). No significant differences were observed between GFZ and 25-HC treatment in STAT1^−/−^ cells (*p* > 0.99). Direct comparison of treated STAT1^−/−^ cells to treated, wild-type HEp-2 cells (GFZ, 25-HC) revealed significantly increased syncytia formation in both treatments for STAT1^−/−^ cells. No significant differences were observed in the syncytia size of treated STAT1^−/−^ cells compared to those of untreated, infected wild-type HEp-2. Notably, differences in the kinetics of syncytia formation were observed between 25-HC and GFZ treatment for both wild-type and STAT1^−/−^ cells. While syncytia size increased throughout the experiment in GFZ-treated cells, syncytia size increased rapidly upon 25-HC treatment, peaking at 36 hpi, followed by a rapid decline afterwards.

### The role of cholesterol in the transport and stability of RSV F protein

**Fig. 6:**
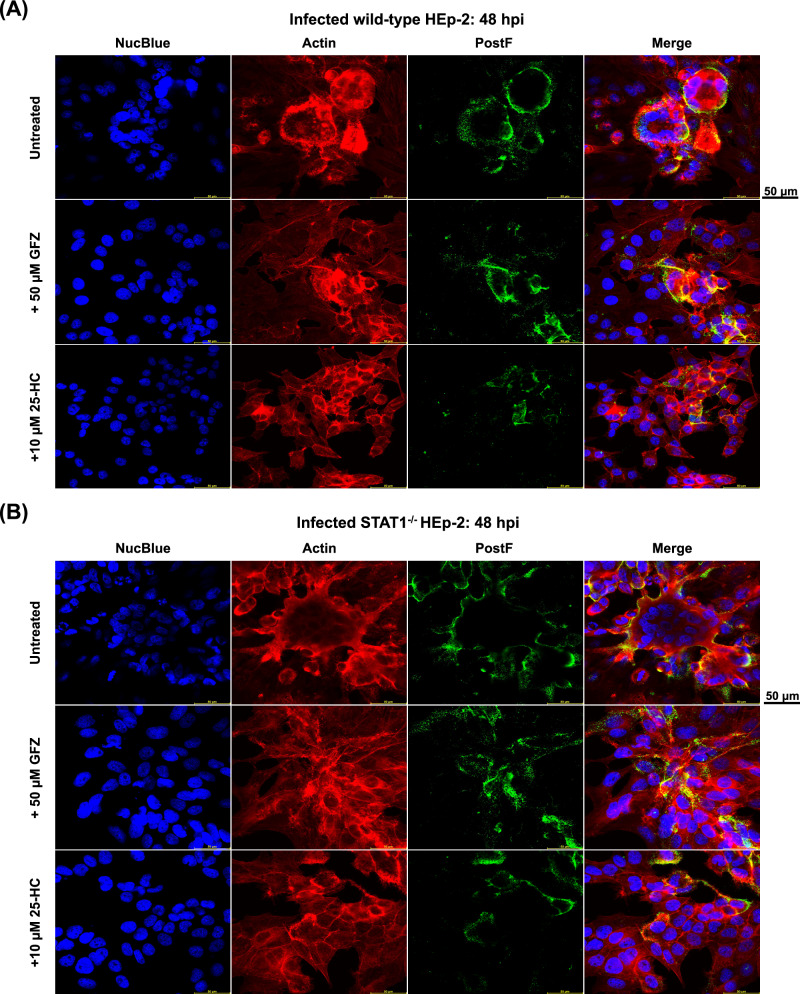
Surface expression of RSV PostF protein. Confocal microscopy images of RSV-A-0594-infected wild-type (**A**) and STAT1^−/−^ (**B**) HEp-2 cells (MOI 0.05) at 48 hpi. Infected cells were left untreated (top), treated with 50 μM Gemfibrozil (GFZ, middle), or 10 μM 25-hydroxycholesterol (25-HC, bottom). Cells were stained for RSV postfusion F protein on the cell surface. Intracellular actin was visualized by ActinRed staining and nuclei by NucBlue staining. Images representative of three independent experiments (two technical replicates). Scale bar indicated next to images.

**Fig. 7:**
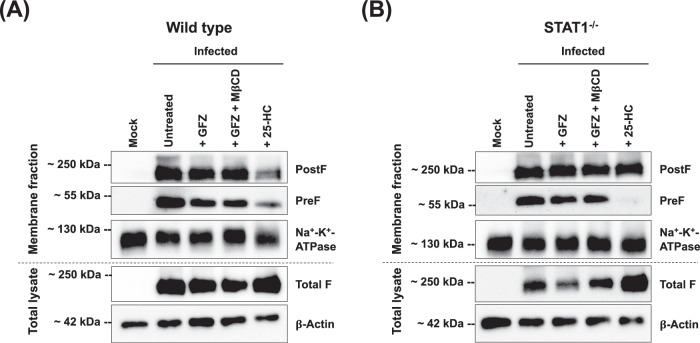
Integration of viral fusion protein into host cellular membranes. Western blot of membrane fractions and total cell lysates in uninfected (‘Mock’) and rRSV-A-0594-eGFP-infected (MOI 0.5) wild-type (**A**) and STAT1^−/−^ HEp-2 (**B**) HEp-2 cells at 48 hpi. Infected cells were left untreated, treated with 50 μM Gemfibrozil (GFZ), treated with 5 μM methyl-β-cyclodextrin (MβCD) and 50 μM GFZ, or with 10 μM 25-hydroxycholesterol (25-HC). Membrane fractions were stained for prefusion F protein, postfusion F protein, and Na^+^-K^+^-ATPase (loading control). Total cell lysates were stained for total F protein (prefusion and postfusion conformation) and β-Actin (loading control). Images representative of three independent experiments.

Fundamental to RSV dissemination and syncytia formation is the transport of the viral F protein to the cell surface and its integration into the membrane. To determine if the distribution of the F protein is affected by changes in intracellular cholesterol levels, we performed immunofluorescence staining targeting the F protein in the PostF conformation on the surface of rRSV-A-0594-eGFP-infected wild-type and STAT1^−/−^ HEp-2 cells, both with and without cholesterol-lowering drugs (Fig. 6). In untreated, infected wild-type HEp-2 cells, the PostF protein appeared evenly distributed on the cell surface of individual infected cells, especially on the surface of syncytia (Fig. 6A). This pattern of PostF expression on the cell surface was even more pronounced on infected, untreated STAT1^−/−^ HEp-2 cells, with PostF forming an even and intense border along large syncytia that distinguished them from uninfected, neighboring cells (Fig. 6B). The even distribution of PostF observed in untreated wild-type and STAT1^−/−^ RSV-infected HEp-2 cells was disrupted after treatment with GFZ and 25-HC. Confocal imaging revealed a bulging morphology of RSV-induced syncytia in HEp-2 cells, resulting in minor variations in apparent nuclear size attributable to Z-stack focus positioning rather than treatment effects or imaging inconsistencies. The corresponding images of uninfected, treated wild-type and STAT1^−/−^ HEp-2 cells are shown in Supplementary Fig. 13. Having demonstrated that intracellular cholesterol levels influence the distribution and abundance of the RSV F protein, we aimed to analyze the integration of the F protein into cellular membranes in the absence or presence of cholesterol-lowering agents. We performed western blot analysis on rRSV-A-0594-eGFP-infected wild-type and STAT1^−/−^ HEp-2 cells, targeting the PreF and PostF proteins in the membrane fractions, as well as total F protein in whole cell lysates (Fig. 7). Western blots showed visually comparable levels of PreF and PostF in the membrane fractions of infected, untreated cells, which were similar between wild-type (Fig. 7a) and STAT1^−/−^ HEp-2 (Fig. 7b) cells. Levels of total F in whole cell lysates, however, were markedly reduced in STAT1^−/−^ HEp-2 cells compared to wild-type HEp-2 cells. Upon treatment with GFZ or GFZ in combination with MβCD, a slight increase in PostF was observed in membrane fractions of both wild-type and STAT1^−/−^ HEp-2, while PreF total F were comparable or slightly reduced compared to untreated controls. This change was also visible upon treatment of infected wild-type and STAT1^−/−^ HEp-2 cells with 25-HC. While a band for PreF was still visible in wild-type HEp-2 cells, no PreF was detectable in the membrane fraction of STAT1^−/−^ HEp-2 cells. Notably, levels of total F protein in whole cell lysates were markedly increased in both cell types upon 25-HC treatment.

## Discussion

STAT1 is widely recognized as a key regulator of IFN-driven antiviral immunity^19,46,47^. Beyond its canonical role in promoting ISG expression, growing evidence indicates that STAT1 also directly influences cellular metabolic pathways, including cholesterol and lipid biosynthesis^20,48^. To address this paradigm in the context of RSV infections, we generated STAT1-knockout cells derived from HEp-2 lung epithelial-like cells. Having confirmed the knockout on genome and protein level, alterations in cell morphology were evident, while no differences in cellular growth dynamics were observed, indicating a link between STAT1 signaling and cellular membrane properties, particularly cholesterol as a major lipid component of mammalian membranes^49,50^. Our transcriptomics data reveal that STAT1 is not only involved in classical antiviral host responses but also acts as an essential modulator of the SREBP-SCAP-INSIG axis, thereby maintaining cholesterol homeostasis during RSV infection.

The main components of this axis are the transcription factors SREBP-1 (with isoforms SREBP-1a and SREBP-1c) and SREBP-2, encoded by *SREBF1* and *SREBF2*, respectively^30^. Although all SREBPs can regulate the transcription of their targets, SREBP-1 is primarily involved in regulating fatty acid synthesis, whereas SREBP-2 preferentially regulates the expression of genes related to cholesterol biosynthesis and influx, including *HMGCS*, *HMGCR*, and *LDLR*^30^. Transcriptional activity of SREBPs depends on the escort protein SCAP and feedback inhibitors INSIG-1/2, which act as direct sensors for sterols and oxysterols, respectively^29,51,52^. High abundance of cholesterol results in the interaction of SCAP with INSIG, which hinders the integration of the SREBP-SCAP complex into export vesicles, thereby retaining it in the ER and restricting further activation of cholesterol biosynthesis^33,51,53^. Transcription of *SREBF1/2* and *INSIG1* is regulated in a feed-forward loop, in which SREBPs simultaneously activate *INSIG1* and their own gene expression^30^, while transcription of *SCAP* and *INSIG2* is independent of SREBP^52,54^. Moreover, high intracellular levels of sterols activate Liver X Receptor (LXR) transcription factors, which in turn regulate intracellular cholesterol and lipid levels by transcriptional regulation of genes involved in cholesterol biosynthesis (*SREBF*), influx (*IDOL*), and efflux (*ABCA1*, *ABCG1, SCARB1*)^55–58^. In the present study, *STAT1* deletion resulted in impaired transcriptional regulation of the SREBP-SCAP-INSIG axis, evident by reduced expression of *SREBF2*, *SCAP*, and *INSIG1*. As a consequence of reduced *SREBF2* transcription, expression of genes involved in cholesterol biosynthesis (e.g., *HMGCR*, *HMGCS*, *MVK*, *IDI1*, *SQLE*) and cholesterol influx (*LDLR*, *LDLRAP1*) were downregulated, while LXR-encoding genes (*NR1H2/3*) and LXR targets were upregulated (*ABCG1*, *SCARB1*, *IDOL*), indicating reduced cholesterol biosynthesis and increased cholesterol efflux as a response to high intracellular sterol levels. Notably, genes involved in cholesterol esterification (*SOAT1*) were also downregulated in STAT1^−/−^ cells. As the efficient regulation of cholesterol metabolism by SREBP-2 and INSIG-1 is disrupted upon STAT1 deletion, compensatory activation of *SREBF1* and *INSIG2* transcription may occur to maintain some regulation of cholesterol metabolism. Collectively, these findings indicate that, in the absence of STAT1 regulation, SREBP2-mediated transcription, efficient cholesterol efflux, and esterification are impaired, leading to intracellular accumulation of free cholesterol.

Upon infection, intracellular free cholesterol levels in both wild-type and STAT1^−/−^ cells decrease, indicating redistribution of free cholesterol through increased efflux and esterification. In STAT1^−/−^ cells, this was shown by elevated transcription of *ABCG1* and *ACAT2*. In response to low intracellular cholesterol, the SREBP-SCAP-INSIG axis was reactivated, and key cholesterol biosynthesis genes were upregulated in STAT1^−/−^ cells. This stands in contrast to the canonical STAT1-driven response in wild-type HEp-2 cells, where transcription of these genes is suppressed in response to infection, thereby limiting resources available for viral dissemination. This dysregulation in cholesterol metabolism altered cell membrane properties and may ultimately promote RSV-mediated syncytia formation. Through supplementation of 25-HC, regulation of SREBP was restored, and syncytia formation in STAT1^−/−^ cells was restricted, underscoring the dependency of syncytia formation on intracellular cholesterol levels. This increased formation of syncytia appears to be independent of viral replication, as no significant differences in viral titers were observed between wild-type and STAT1^−/−^ HEp-2 cells, suggesting that syncytia formation primarily reflects viral cytotoxicity rather than spread. As the data were obtained in a highly susceptible, cancerous cell line, further experiments will be necessary to address whether syncytia facilitate viral spread or primarily represent a cytopathic effect of infection and therefore contribute to disease severity, as indicated by previous studies^41^.

Our study complements previous studies that dissected the role of cholesterol metabolism during RSV infections, showing that cholesterol-rich lipid rafts are essential for RSV entry into human bronchial epithelial cells^59^. RSV was shown to alter cholesterol metabolism by interfering with intracellular cholesterol transport, thereby activating the SREBP2-Low-Density Lipoprotein Receptor (LDLR) axis and leading to the accumulation of cholesterol in lysosomes^25^. These microdomains serve as a storage site for the RSV F protein, and enrichment of the viral F protein was found to be cholesterol-dependent, which is supported by the presence of two cholesterol recognition amino acid consensus sites in the ectodomain of the F protein^25,60^. Consistent with these findings, we demonstrate that in the presence of cholesterol-lowering 25-HC, a natural inhibitor of the SREBP-SCAP axis, most of the viral F protein accumulated in the cytosol compared to untreated cells, while syncytia formation was impaired. A reduction in infection levels and, consequently, F protein surface expression was also observed upon 25-HC treatment of STAT1^−/−^ and wild-type HEp-2 cells, as shown by confocal imaging. Further, membrane-associated F protein was predominantly abundant in its postfusion conformation, which is no longer capable of mediating membrane fusion^61^. Cholesterol and cholesterol-rich lipid rafts may therefore be required for stabilizing the F protein in its prefusion conformation, ensuring efficient trafficking to the surface in a fusion-competent state. This is supported by previous studies showing that RSV infection in the presence of cholesterol-lowering drugs resulted in less stable progeny virus^62^. Similar results were observed upon addition of GFZ and MβCD to STAT1^−/−^ cells, although not to the same extent as observed in 25-HC-treated cells, indicating different mechanisms that result in lower cholesterol levels. GFZ is a PPARα agonist, and its addition to cells leads to increased fatty acid oxidation, altered lipid metabolism, and, indirectly, increased cholesterol trafficking^63^. MβCD, on the other hand, transiently depletes cholesterol from membranes by forming inclusion complexes with cholesterol^64^. Therefore, while GFZ and MβCD reduce cholesterol levels indirectly or transiently, 25-HC reduces long-term cellular cholesterol levels by inhibition of cholesterol synthesis. This was also observed in Filipin III staining, where no significant difference was observed between GFZ-treated and untreated infected cells. As GFZ is widely known as a cholesterol-lowering agent^65^, these findings indicate that although GFZ did not affect surface cholesterol, it may have reduced intracellular cholesterol levels, thereby reducing syncytia formation. Likewise, MβCD-mediated cholesterol depletion was reversed shortly after treatment. Only for 25-HC, a time-dependent decrease in total cholesterol was visible.

Our findings have significant implications for individuals with impaired immune systems, i.e., infants, immunocompromised individuals, and older adults, who are at higher risk of severe RSV infections. Altered interferon and STAT1 signaling, especially in the context of an immature immune system, genetic mutations, or immunosenescence, are major risk factors for severe RSV infections^66–68^. Interestingly, STAT1 activity is increased in older adults in the context of ‘inflammaging’, i.e., chronic, low-grade inflammation, which interferes with lipid metabolic regulators^69,70^. As a consequence, dyslipidemia is regularly observed in older adults, which may provide a basis for efficient RSV infection and spread^71^. Although studies linking impaired interferon signaling, increased cholesterol accumulation, and viral disease severity are limited, our findings broaden the clinical understanding of severe RSV infections in these at-risk groups by providing a mechanistic link between impaired STAT1 and, consequently, interferon signaling, altered cholesterol metabolism, and the increased formation of syncytia, a hallmark of severe RSV infections^41^. Notably, STAT1 deficiency not only resulted in increased syncytia formation but also increased IC_50_ for Presatovir, a small molecule inhibitor blocking the conformational changes of the F protein, thereby preventing fusion with the host membrane^72^. Considering that Presatovir targets the viral protein, and complete fusion inhibition was observed in both wild-type and STAT1^−/−^ cells, this suggests that the underlying mechanism of cell-to-cell fusion applies to both cell types, and that host factors are responsible for the observed differences. Reduced drug efficacy may result from increased accumulation of viral F protein on the cell surface or an altered composition of the viral membrane due to higher cholesterol content. Further in vivo studies will be required to address this link and its relevance for the therapeutic treatment of this age group. Nonetheless, our findings underscore the need for integrated therapeutic strategies that target both viral proteins and host cholesterol homeostasis, particularly in individuals at risk.

While our study provides novel insights into the role of STAT1 in regulating cholesterol metabolism and RSV-mediated syncytia formation, it also has some limitations. Using in vitro cell culture models, we gained a precise understanding of the molecular mechanisms involved. Further in vivo studies will be required to confirm our observations in the context of a more complex microenvironment during RSV infections. While our findings indicate that cholesterol-dependent F protein trafficking and distribution underlie increased syncytia formation, the precise mechanisms contributing to this phenotype remain incomplete and require further research. Finally, while we identified a crucial link between STAT1 signaling and cholesterol metabolism, other antiviral pathways may be of interest for comprehensive profiling of protective and harmful immune responses during severe RSV infections. These considerations provide important directions for future studies to validate and extend our understanding of STAT1-dependent regulation of cholesterol metabolism in severe cases of RSV infection.

In conclusion, our study integrates and advances the understanding of STAT1-dependent transcriptional regulation of the SREBP-SCAP pathway in maintaining cholesterol homeostasis and its role in controlling RSV-induced syncytium formation. STAT1 deficiency disrupts this balance, leading to dysregulated cholesterol biosynthesis and trafficking, which results in increased fusion and viral spread. These findings provide novel insights into immunometabolic processes during RSV infections and may aid the development of alternative therapeutic strategies that target host cholesterol metabolism, in addition to antiviral treatment. As older adults are at high risk for severe RSV infections, and a large proportion of this age group has high Low-Density Lipoprotein (LDL) cholesterol levels and hypercholesterolemia^71^, we identified a mechanism that could explain this increased risk based on age-related changes in immune signaling and cholesterol metabolism. While the majority of older adults are treated with statins or other cholesterol-lowering drugs to reduce the risk of cardiovascular diseases^73^, our findings suggest that this also may decrease the risk of severe RSV infections in this age group.

## Methods

### Cells

HEp-2 (ATCC, CCL-23) and STAT1 knockout (STAT1^−/−^) HEp-2 cells were cultured in Minimum Essential medium with Earl’s salts (Capricorn Scientific, MEM-A) supplemented with 10% fetal bovine serum (FBS, Gibco), 1% penicillin/streptomycin (P/S, Capricorn Scientific), 1% non-essential amino acids (Gibco), and 1% GlutaMAX (Gibco) at 37 °C, 5% CO_2_. Cells were passaged at 80–90% confluency.

### Generation of STAT1-KO cell line

STAT1^−/−^ cells were generated by transfection of the pSpCas9(BB)-2A-Puro V2.0 plasmid into HEp-2 cells (ATCC, CCL-23). The plasmid is based on a PX459 backbone (Addgene, Plasmid ID 62988, deposited by Feng Zhang), which includes genes encoding the Cas9 protein from *Streptococcus pyogenes*, as well as genes for ampicillin and puromycin resistance for selection in bacteria and mammalian cells, respectively^74^. Single-guide RNAs (sgRNAs) were designed using the ccTop tool^75^, targeting the first exon of the human *STAT1* gene (Transcript no. ENST00000361099.8; target sequence 5’-ATTGGGCGGCCCCCCAATACAGG-3’). sgRNAs were chosen based on a high CRISPR score ( > 0.75) and minimal ( < 20) off-target effects^76^. DNA strands complementary to the chosen sgRNAs were ordered (Microsynth) and used for cloning into the pSpCas9(BB)-2A-Puro V2.0 plasmid. The following sequences were used: 5’-CACCGATTGGGCGGCCCCCCAATAC-3’ (forward) and 3’-CTAACCCGCCGGGGGGTTATGCAAA-5’ (reverse). Oligo pairs were annealed and phosphorylated using a T4 polynucleotide kinase (NEB) according to the manufacturer’s instructions. pSpCas9(BB)-2A-Puro was digested with BbsI (NEB) and dephosphorylated using calf intestine alkaline phosphatase (NEB) according to the manufacturer’s instructions. The phosphorylated sgRNA was then ligated into the digested backbone using T4 ligase (NEB), transformed into competent DH5α *E. coli* (Zymo Research), and plated on carbenicillin-containing LB agar plates. Successful cloning of sgRNAs into the vector was confirmed by Sanger sequencing (Microsynth). Plasmids were then transfected into HEp-2 cells using ViaFect transfection reagent (Promega) according to the manufacturer’s instructions. pSpCas9(BB)-2A-GFP (PX458) (Addgene, Plasmid ID 48138, deposited by Feng Zhang) served as transfection control. Cells were cultured in puromycin-containing growth medium (1 μg/ml) 24 h (h) post-transfection, and medium refreshed every two days. Cells were cultured until >50% confluency was reached. Single cells were manually prepared and cultured in HEp-2 growth medium with BriClone Hybridoma Cloning supplement (Dublin City University) in a 1:1 ratio until 70% confluency of clonal cultures was reached. Cells were subsequently cultured in HEp-2 growth medium.

### Confirmation of STAT1 knockout

Genomic DNA of wild-type HEp-2 and STAT1^−/−^ clones was isolated using the Monarch® Spin gDNA Extraction Kit (NEB) according to the manufacturer’s instructions. A PCR targeting the *STAT1* gene was performed using the AmpliTaq Gold™ DNA Polymerase kit (Applied Biosystems) according to the manufacturer’s instructions. Sequences of the forward and reverse primers used for PCR were 5’-ACACCCCATGGTACAGGAGT-3’ and 5’-GCTTAGAGCCCCAGTTGAGA-3’, respectively. STAT1 knockout was confirmed at the genomic level by Oxford Nanopore Sequencing service (Microsynth) and at the protein level by Western blot of total cell lysates. To this end, HEp-2 cells were lysed with M-PER™ Mammalian Protein Extraction Reagent (Thermo Fisher Scientific) according to the manufacturer’s instructions. Total protein concentration was quantified by Quick Start Bradford Protein Assay (Bio-Rad) according to the manufacturer’s instructions. 5 μg of total protein was loaded onto a 10% SDS-PAGE under reducing conditions using 1x Lane Marker Reducing Sample Buffer (Thermo Fisher Scientific) and transferred to an Immobilon®-PSQ PVDF membrane (Merck) using a Trans-Blot® Turbo™ Transfer System (Bio-Rad). Membranes were blocked with 5% skimmed milk powder in Tris-buffered saline containing 0.05% Tween-20 (TBS-T) for 1 h at room temperature (RT), followed by incubation in primary antibodies targeting STAT1 (STAT1-79, 0.5 μg/mL, Thermo Fisher Scientific) or β-Actin (BA3R, 0.1 μg/mL, Thermo Fisher Scientific) at 4 °C overnight. Membranes were washed with TBS-T and incubated with HRP-conjugated goat anti-mouse IgG antibody (A16072, 0.5 μg/mL, Invitrogen) for 1 h at RT. Signals were detected using SuperSignal West Pico PLUS chemiluminescent substrate (Thermo Fisher Scientific) and visualized using a ChemiDoc MP imaging system (Bio-Rad).

### Viruses and viral infections

Infection of cells was performed using either the contemporary RSV-A-0594 (ON1 genotype) strain or recombinant (r) RSV-A-0594-eGFP reporter strain as described in ref. ^77^. Virus stocks were generated by infecting HEp-2 cells at 60-80% confluency in Opti-MEM supplemented with 1% P/S (Gibco). Cells were incubated at 37 °C, 5% CO_2_ until virus-induced cytopathic effects were visible (3–5 days post-infection). Cells were scraped off and whole-cell suspensions were centrifuged to remove cell debris (1000 × g, 10 min, 4 °C). The supernatants were collected, mixed with 50% polyethylene glycol (PEG, Carl Roth) 6000 to a final concentration of 10%, and incubated at 4 °C for 4 h. Samples were centrifuged (3000 x g, 30 min, 4 °C) and virus resuspended in Halt’s balanced salt solution (Gibco) containing 20% sucrose. Virus suspension was aliquoted, snap-frozen in liquid nitrogen, and stored at −150 °C. Viral titers were determined by a standard 50% tissue culture infectious dose (TCID_50_)/mL assay on HEp-2 cells, as described by Reed and Muench^42^. For all infections, cells were seeded one day prior to infection to allow cell attachment. Cells were infected with the indicated virus and multiplicity of infection (MOI) and diluted in Opti-MEM + 1% P/S. Cells were incubated at 37 °C, 5% CO_2_ for 2 h, inoculum removed, and cells washed twice with phosphate-buffered saline (PBS, Capricorn Scientific) to remove residual virus. Cells were then maintained in Opti-MEM + 1% P/S for the duration of the experiment.

### Replication kinetics

Wild-type and STAT1^−/−^ HEp-2 cells were infected with RSV-A-0594 and rRSV-A-0594-eGFP (MOI 0.05) to assess differences in viral replication kinetics between the two cell types. Infected cells were incubated at 37 °C, 5% CO_2_, and samples collected at 0, 24, 48, 72, 96, and 120 h post-infection (hpi). Cells and supernatants were collected, virus particles precipitated from clarified supernatants by PEG 6000, and samples stored at −150 °C as described above. Titers for RSV-A-0594 were visualized by immunostaining. To this end, plates were fixed at 5 days post-infection with 4% paraformaldehyde (PFA) in PBS and permeabilized with 0.5% Triton X-100. Cells were blocked in PBS containing 5% bovine serum albumin (BSA, Carl Roth) and incubated with polyclonal goat anti-RSV (AB1128, 1:500, Merck) for 1 h at RT. Horseradish peroxidase (HRP)-conjugated donkey anti-goat polyclonal antibody (AB6885, 2 μg/mL, Abcam) was used as a secondary antibody, and staining visualized using TrueBlue peroxidase substrate (SeraCare). For rRSV-A-0594-eGFP, viral titers were determined using a Leica DM8 fluorescence microscope.

### Fusion inhibition assay

For viral fusion inhibition assays, wild-type and STAT1^−/−^ HEp-2 cells were seeded into black-walled, clear-bottomed plates and infected with rRSV-A-0594-eGFP (MOI 0.05) the following day. Serial two-fold dilutions of Presatovir (MedChemExpress) were prepared in Opti-Mem + 1% P/S, ranging from 0.4 nM to 250 nM, and directly added to cells in the presence of virus. Infected, untreated HEp-2 cells served as a control. Cells were incubated at 37 °C, 5% CO_2_ for 72 h. The fluorescence intensity of the eGFP signal was measured using a Tecan Spark microplate reader (Excitation/Emission: 480/520 nm).

### Quantification of free cholesterol

Levels of free cholesterol were quantified in wild-type and STAT1^−/−^ HEp-2 cells during homeostasis and upon infection or stimulation with IFNs. Cells were seeded into white-walled, white-bottomed tissue culture plates at a concentration of 1 × 10^4^ cells/mL, and infected with RSV-A-0594 (MOI 0.05 and 0.5) or stimulated with IFN-α2 (100 ng/mL, MedChemExpress) and IFN-γ (50 ng/mL, Thermo Fisher Scientific). Untreated, uninfected cells served as negative controls. To confirm assay validity, cholesterol was depleted from cells by methyl-β-cyclodextrin (MβCD, 5 mM, MedChemExpress) 30 min before cholesterol levels were measured. MβCD was removed, cells rinsed with PBS, and Opti-Mem + 1% P/S was added to the cells. Free cholesterol levels were quantified using the Cholesterol/Cholesterol Ester-Glo™ Assay (Promega) according to the manufacturer’s instructions. Cholesterol levels were quantified at 0 (untreated only), 24, and 48 hpi. Luminescence was measured using a Tecan Spark multiplate reader.

### RNA-Seq

Gene expression of STAT1^−/−^ HEp-2 was compared to wild-type HEp-2 by RNA-Seq using Novogene’s Illumina NovaSeq X Plus Series (PE150) platform. Cells were infected with rRSV-A-0594-eGFP (MOI 0.05) and RNA isolated at 0, 12, 24, and 48 hpi using a Monarch® Spin RNA Isolation Kit (NEB). Data were analyzed using the DESeq2 method (LRT test) with RaNA-Seq, a cloud-based RNA-Seq analysis platform^78^. Data was visualized using Python (v3.13.5) with Matplotlib and Seaborn libraries. Gene boundaries for viral transcripts are listed in Supplemental Table 1.

### Western blots of membrane-bound and total F protein

To address differences in the amount of membrane-bound and total F protein in infected wild-type and STAT1^−/−^ HEp-2 cells, cellular membrane fractions and total cell lysates were analyzed by western blotting after infection with rRSV-A-0594-eGFP (MOI 0.5). To determine the effect of cholesterol-lowering drugs on F protein membrane integration, infected cells were treated with 50 μM gemfibrozil (GFZ, MedChemExpress), 50 μM GFZ combined with 5 μM MβCD, or 10 μM 25-HC (MedChemExpress). MβCD was added to cells for 30 min in the presence of the virus and removed with the inoculum. GFZ and 25-HC were added to cells after infection and remained in the well until sample collection. Lysates were collected at 48 hpi using Mem-PER™ Plus Membrane Protein Extraction Kit (Thermo Fisher Scientific) or M-PER™ Mammalian Protein Extraction Reagent (Thermo Fisher Scientific) according to the manufacturer’s instructions. Protein concentration was measured by Quick Start Bradford Protein Assay (Bio-Rad) according to the manufacturer’s instructions. Samples were prepared either under non-reducing conditions (for post-fusion F (PostF) and total F detection), using Pierce™ Lane Marker Non-Reducing Sample Buffer (Thermo Fisher Scientific), or reducing conditions (for pre-fusion F (PreF) and loading controls), using Pierce™ Lane Marker Reducing Sample Buffer (Thermo Fisher Scientific). Total protein separation (5 μg) was performed by SDS-PAGE, followed by Western blotting, as described above. Primary antibodies targeting PreF (Nirsevimab, 4 μg/mL, Thermo Fisher Scientific), PostF (131-2 A, 1 μg/mL, Merck), total F (Palivizumab, 8.4 μg/mL, MedChemExpress), Na^+^-K^+^-ATPase (ST0533, 20 ng/mL, Thermo Fisher Scientific), or β-Actin (BA3R, 0.1 μg/ml, Thermo Fisher Scientific), as well as HRP-conjugated, secondary antibodies goat anti-mouse IgG antibody (A16072, 0.5 μg/mL, Invitrogen), donkey anti-human IgG antibody (ab102410, 1 μg/mL, Abcam), or goat anti-rabbit IgG antibody (ab6721, 0.4 μg/mL, Abcam) were used. Membranes were detected using SuperSignal West Pico PLUS chemiluminescent substrate (Thermo Fisher Scientific), and images visualized using a ChemiDoc MP system (Bio-Rad).

### Live cell imaging

Live-cell imaging was performed using an IncuCyte S3 system (Sartorius). For visualization and quantification of cellular expansion, wild-type or STAT1^−/−^ HEp-2 cells were seeded into 96-well plates at 2.5 × 10^3^ cells/well and incubated for live-cell imaging once cells attached to wells. Cells were maintained in standard growth medium, and phase-contrast images taken every 3 h for 4.5 days. To quantify cell eccentricity, cell-by-cell analysis was performed using IncuCyte S3 2021 C software. To exclude dead cells from analysis, a minimum area of 600 μm^2^, a minimum eccentricity of 0.3, and a Hole fill of 1000 μm^2^ were set. To avoid cell overlap, cell eccentricity was assessed at approximately 60% confluence. For quantification of syncytia size, wild-type and STAT1^−/−^ HEp-2 cells were seeded into a black-walled, clear-bottom plate and infected with rRSV-A-0594-eGFP (MOI 0.05). After infection, cells were incubated in Opti-Mem + 1% P/S supplemented with 50 μM GFZ, 10 μM 25-HC, or without supplements. Uninfected cells served as negative controls. Plates were incubated for live-cell imaging immediately after removal of inoculum, and wells were imaged every hour using phase contrast and GFP channels. To quantify syncytia size across cell types and treatments, images were analyzed by IncuCyte S3 2021 C software (Sartorius). Cell boundaries were automatically set using the label-free cell-by-cell analyzer (integrated into the software) and adjusted if necessary (Segmentation: Surface Fit, Edge Split On, Hole Fill: 10 μm^2^). Phase images were overlaid with GFP images, and syncytia size was quantified from cell boundaries in the phase images to distinguish infected, non-fused cells from syncytia. A representative image of these overlays is provided in Supplementary fig. 11. Cell debris was excluded from analysis by adjustment of minimum cell size (500 μm^2^) and eccentricity (0.4).

### Surface cholesterol staining

Free cholesterol in wild-type and STAT1^−/−^ HEp-2 cells was visualized using Filipin III. To this end, cells were infected with rRSV-A-0594-eGFP (MOI 0.05). After inoculation, cells were either left untreated or cultured in OptiMem + 1% P/S supplemented with 50 μM GFZ, 50 μM GFZ with 5 mM MβCD (added to the cells 30 min before removal of inoculum), or 10 μM 25-HC. Cells were fixed at 0, 24, and 48 hpi using 4% PFA-PBS. Cells were washed with PBS and stained in a non-permeabilized state with Filipin III (0.05 mg/mL, MedChemExpress) in 10% BSA-PBS for 2 h at RT in the dark to visualize surface cholesterol. Filipin III was removed, and cells were permeabilized with 0.5% Triton X-100 to perform nuclear staining. Intracellular RNA was digested by RNase A (100 μg/mL, Thermo Fisher Scientific) treatment for 30 min at RT. Cellular DNA was then stained with propidium iodide (5 μg/mL, MedChemExpress) for 5 min at RT. Cells were washed three times with PBS and then imaged using a Leica DM8 fluorescence microscope. Quantification of the total-field intensity of Filipin III was performed on raw images using ImageJ2 (v2.16.0/1.54p), followed by background correction. In all images, background correction was performed by subtracting a constant value derived from the cell-free background. Specifically, gray values were measured in cell‑free areas of 10 randomly selected images, and the mean of these values was subtracted from all images. The ‘Corrected Total Cell fluorescence (CTCF)’ was calculated as follows: CTCF = Integrated density – (Area x Mean Gray Value _[Background]_).

### Confocal microscopy

Distribution of PostF protein was visualized by confocal microscopy of wild-type and STAT1^−/−^ HEp-2. Cells were seeded into Nunc™ Lab-Tek™ II CC2™ chamber slides (Thermo Fisher Scientific) and infected with rRSV-A-0504-eGFP (MOI 0.05). After inoculation, cells were cultured in Opti-MEM + 1% P/S without additives or supplemented with 50 μM GFZ or 10 μM 25-HC. Cells were fixed at 48 hpi using 4% PFA-PBS, followed by washing with ice-cold PBS. Cells were blocked with 5% BSA in PBS containing 0.05% Tween-20 (PBS-T) for 1 h at RT, followed by incubation in primary antibody targeting PostF (131-2 A, 10 ng/mL, Merck) for 1 h at RT. Cells were washed with PBS-T and incubated with secondary donkey anti-mouse IgG (H + L) conjugated with Alexa Fluor™ Plus 488 (A32766, 4 μg/mL, Invitrogen) for 1 h at RT. Cells were washed with PBS-T and permeabilized with 0.1% saponin (Roth) for 15 min at RT. Cells were washed with PBS and stained with ActinRed555 (Thermo Fisher Scientific) and NucBlue (Thermo Fisher Scientific) according to the manufacturer’s instructions to visualize actin and nuclei. Cells were washed with PBS again, dried overnight, and mounted with ProLong™ Glass Antifade mounting medium (Invitrogen). Images were acquired using a Leica TCS SP5 confocal microscope.

### Statistical analysis

Statistical analyses were performed using the GraphPad Prism v10.4.1 software. Statistical tests are listed in the figure legend, and a *p*-value of < 0.05 was considered statistically significant for all analyses performed.

## Supplementary information

Supplementary material

Supplementary video1

Supplementary video2

Supplementary video3

Supplementary video4

Supplementary video5

Supplementary video6

Supplementary video7

Supplementary video8

Supplementary video9

Supplementary video10

Supplementary video11

Supplementary video12

## Data Availability

The datasets used in the current study are available within the manuscript and the Supplementary file. Any additional data supporting the findings of this study are available from the corresponding author upon request.
