# Supplementary material for: STAT1 signaling controls cholesterol metabolism in epithelial cells and RSV-induced syncytia formation
Source: Npj Viruses. 2026 Feb 16;4:10. doi: 10.1038/s44298-026-00173-w (PMC12910055; doi:10.1038/s44298-026-00173-w)
Supplement: Supplementary file 1 — Supplementary material [file 44298_2026_173_MOESM1_ESM.pdf]

Supplemental Figure 1:

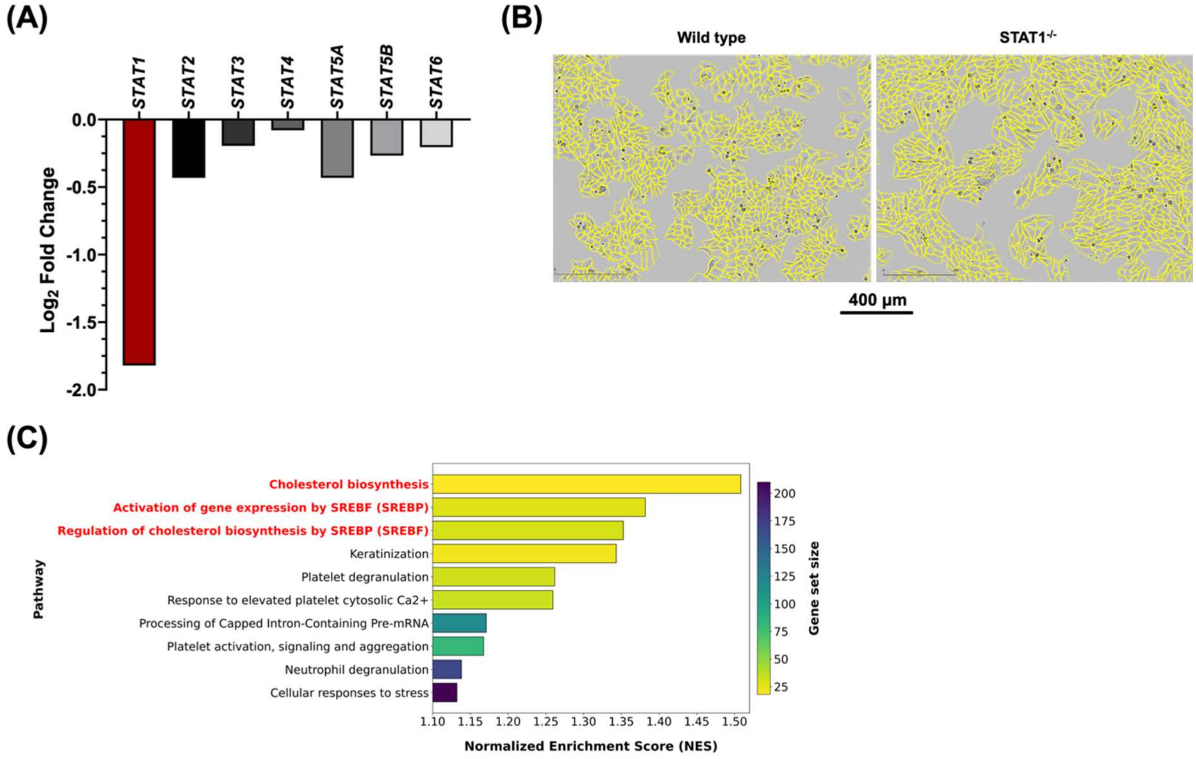

Supplemental Figure 1: Transcriptomic and morphological analysis of uninfected wild-type and STAT1$^{-/-}$ HEp-2 cells. (A) Bar graph indicating Log_2 fold changes of STAT (1-6) expression between wild-type and STAT1$^{-/-}$ HEp-2 cells during homeostatic conditions. **(B)** Bright-field Incucyte images with cell-by-cell analyzer mask (yellow borders) at 72 hours post-seeding for measurement of cell eccentricity. Images are representative of three independent experiments (with 10 replicates each). Scale bar indicated below the images. **(C)** Bar plot of Gene Set Enrichment Analysis of pathways. The plot shows significantly enriched pathways in STAT1$^{-/-}$ cells compared to wild-type HEp-2 cells under homeostatic conditions, based on the Reactome database.

Supplemental Figure 2:

(A)

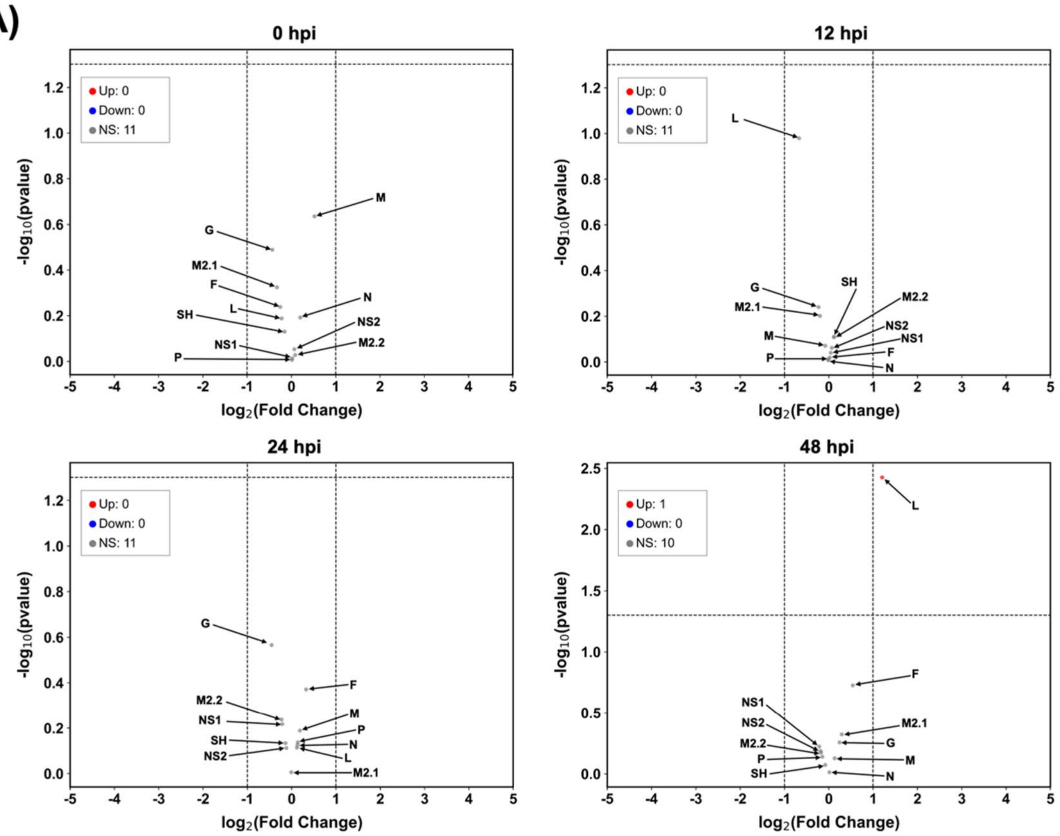

(B)

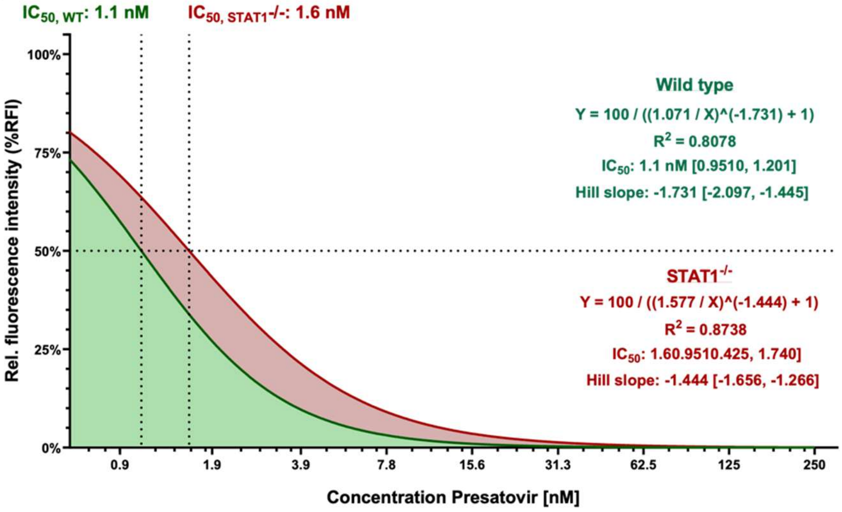

(C)

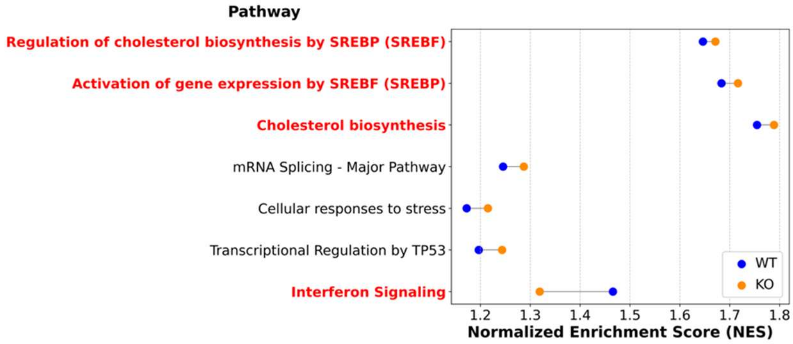

Supplemental Figure 2: Differences in fusion inhibition and transcriptomics. (A) Volcano plots demonstrating the comparison of viral transcript numbers at 0 (top left), 12 (top right), 24 (bottom left), and 48 hpi (bottom right) in STAT1$^{-/-}$ HEp-2 cells compared to wild-type HEp-2 cells with thresholds set at $p < 0.05$ and $|Log_2$ Fold change$| \geq 1$. Arrows indicate transcripts corresponding to viral proteins. **(B)** Non-linear fit of normalized data from Fig. 2d (Presatovir-based fusion inhibition assay) on wild-type (green) and STAT1$^{-/-}$ (red) HEp-2 cells at 72 hpi after infection with rRSV-A-0594-eGFP (MOI 0.05). Relative fluorescence intensity (eGFP) in relation to Presatovir concentration (nM). Dashed lines indicate Half Maximal Inhibitory Concentration (IC_{50}). Graph function, goodness-of-fit value (R^2), IC_{50}, and Hill slope, including 95% confidence intervals (in brackets), are indicated in the graph. Data from three independent experiments, each with eight technical replicates, are shown. **(C)** Cleveland Dot Plot of Gene Set Enrichment Analysis (GSEA) comparing pathways (Reactome database) enriched in both RSV-infected (MOI 0.05) wild-type and STAT1$^{-/-}$ HEp-2 cells based on Normalized Enrichment Scores (NES).

Supplemental Figure 3

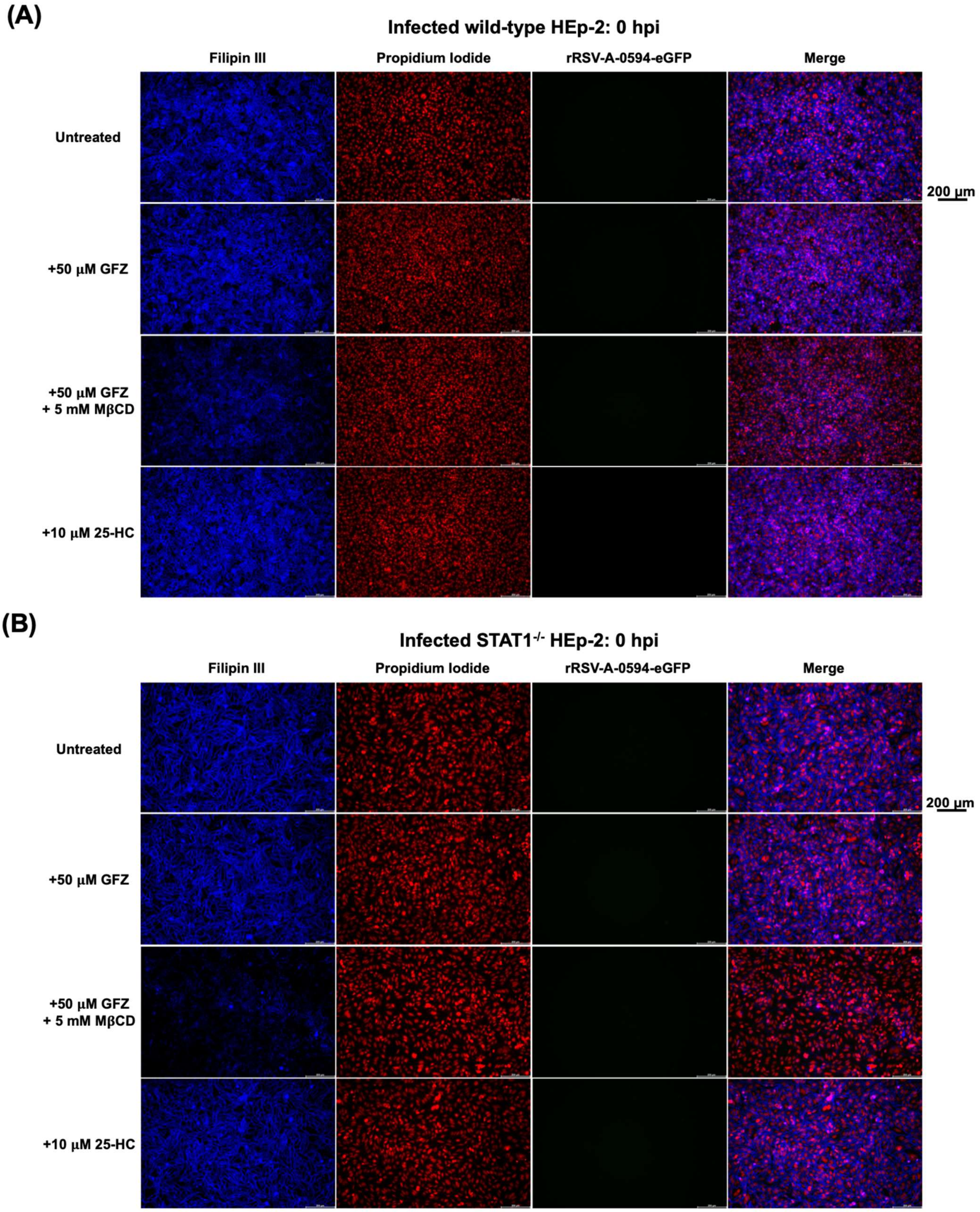

Supplemental Figure 3: Visualization of free cholesterol on cell surface. Representative fluorescence images of rRSV-A-0594-eGFP-infected wild-type **(A)** and STAT1$^{-/-}$ **(B)** HEp-2 cells (MOI 0.05) at 0 hpi. Cells were left untreated (first row), treated with 50 µM Gemfibrozil (GFZ, second row), treated with 5 µM methyl-β-cyclodextrin and 50 µM GFZ (third row), or with 10 µM 25-hydroxycholesterol (25-HC, bottom panel). Free cholesterol in plasma membranes was visualized by Filipin III staining (0.05 mg/ml), and DNA was visualized by Propidium iodide (PI) staining (5 µg/ml). Images representative of three independent experiments. Scale bar indicated next to images.

Supplemental Figure 4:

(A)

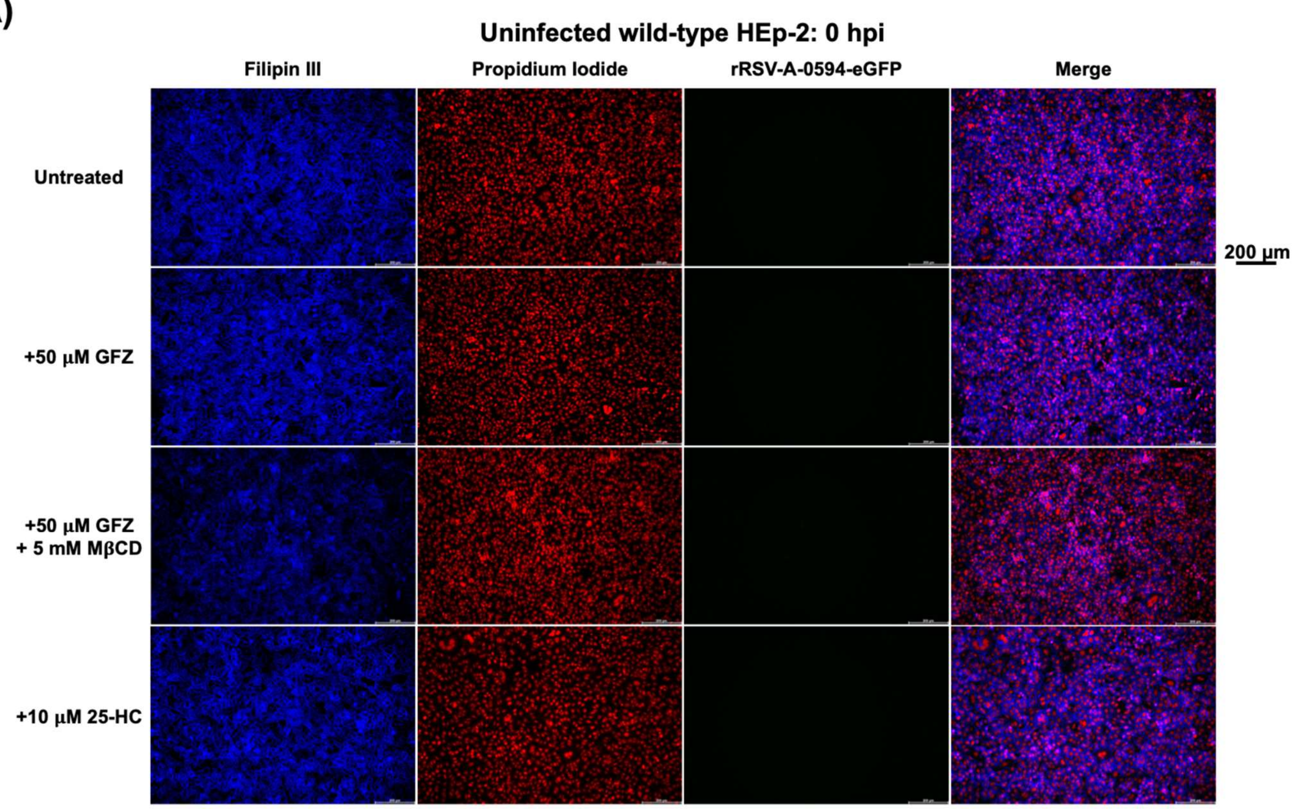

(B)

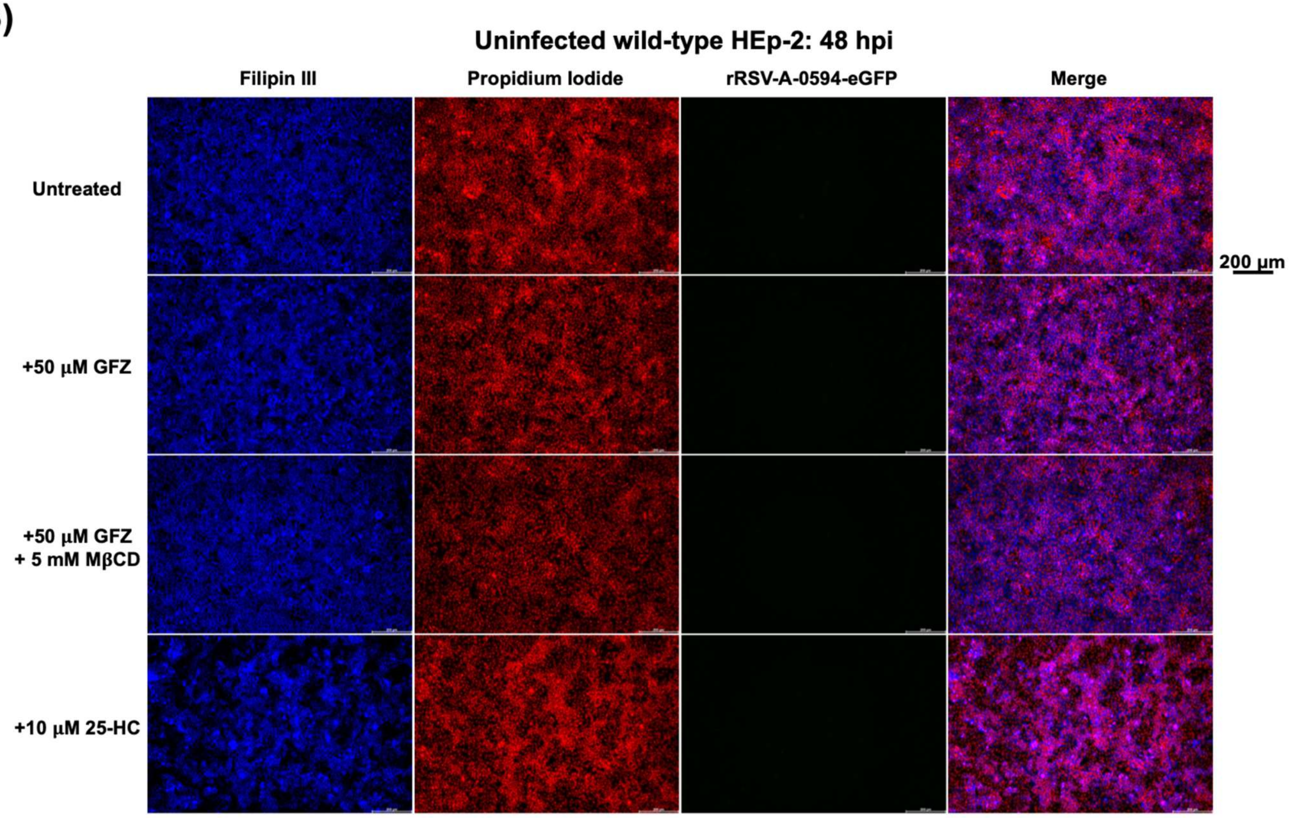

Supplemental Figure 4: Visualization of free cholesterol on cell surface II. Representative fluorescence images of mock-infected wild-type HEp-2 cells (MOI 0.05) at 0 hpi **(A)** and 48 hpi **(B)**. Cells were left untreated (first row), treated with 50 µM Gemfibrozil (GFZ, second row), treated with 5 µM methyl-β-cyclodextrin and 50 µM GFZ (third row), or with 10 µM 25-hydroxycholesterol (25-HC, bottom panel). Free cholesterol in plasma membranes was visualized by Filipin III staining (0.05 mg/ml), and DNA was visualized by Propidium iodide (PI) staining (5 µg/ml). Images representative of three independent experiments. Scale bar indicated next to images.

Supplemental Figure 5:

(A)

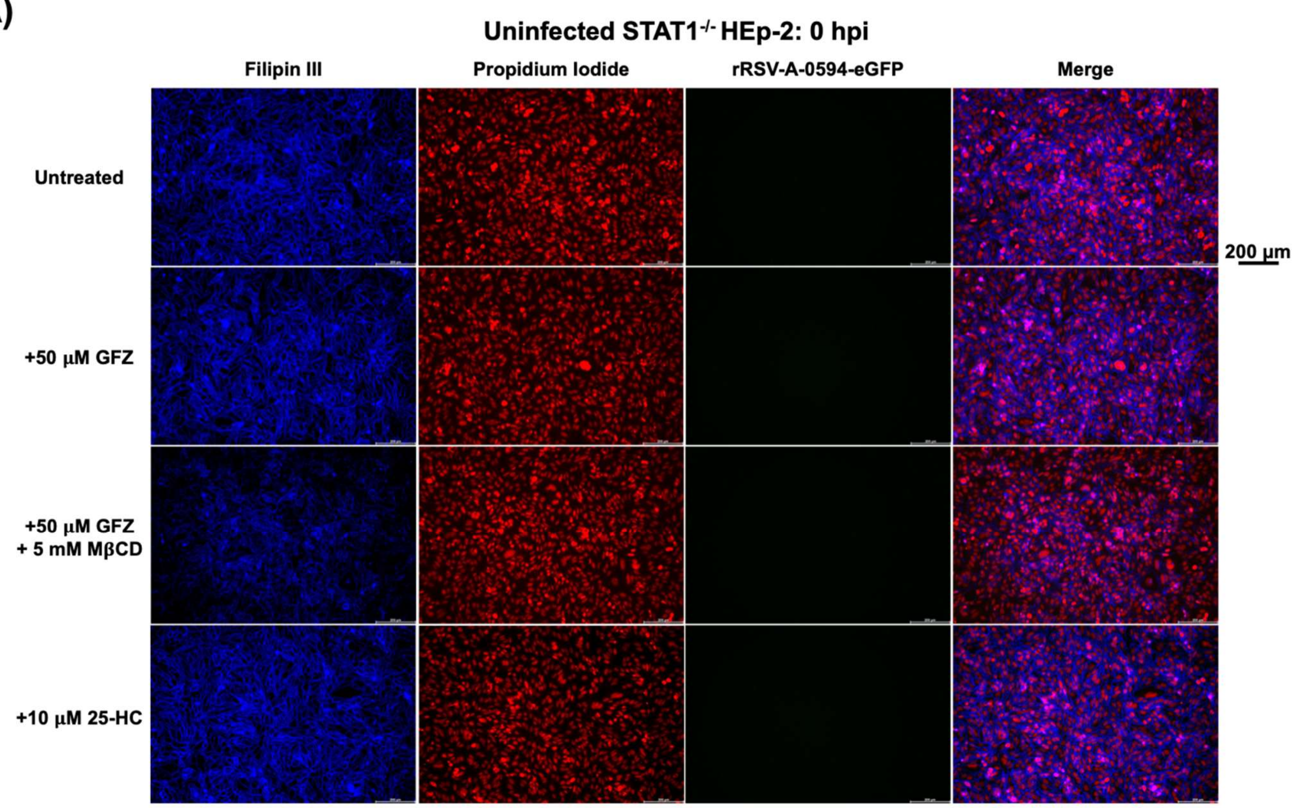

(B)

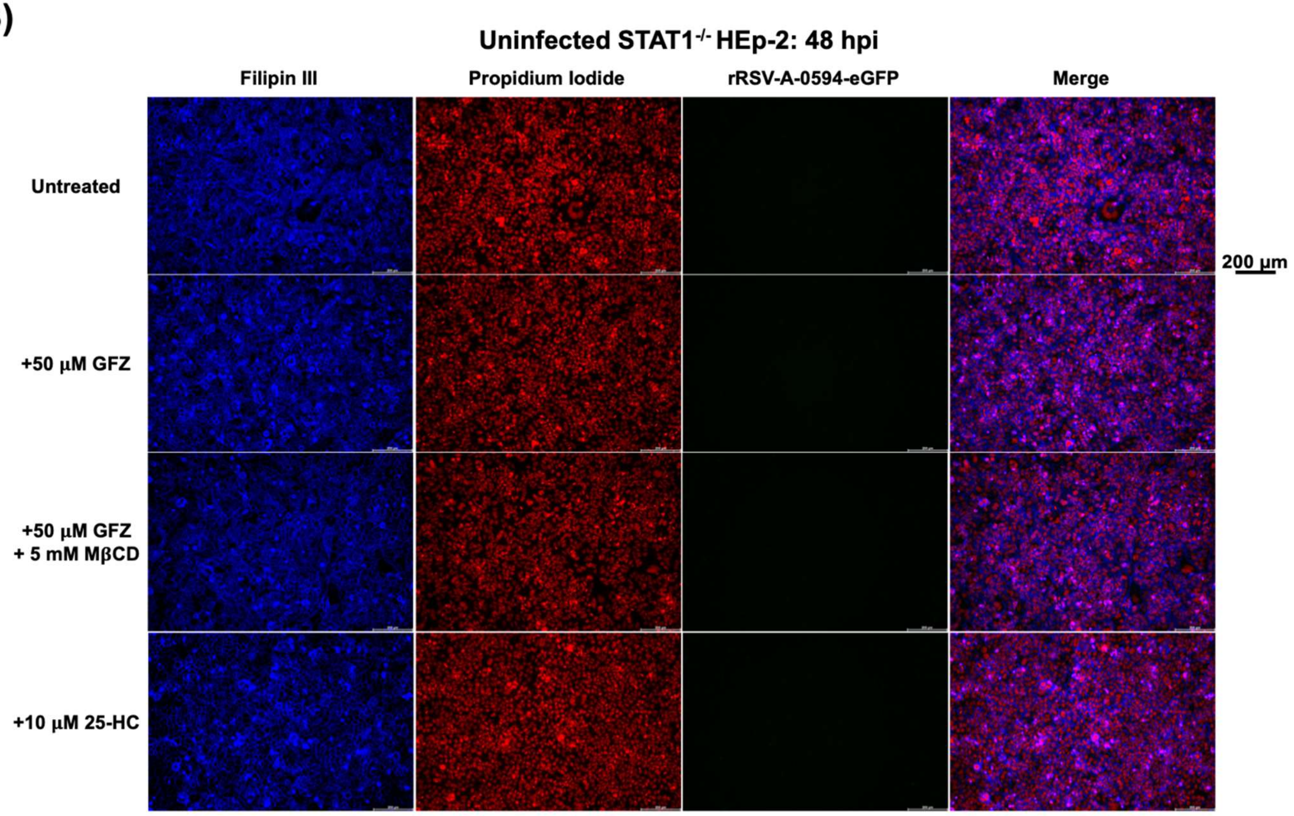

Supplemental Figure 5: Visualization of free cholesterol on cell surface III. Representative fluorescence images of mock-infected STAT1$^{-/-}$ HEp-2 cells (MOI 0.05) at 0 hpi **(A)** and 48 hpi **(B)**. Cells were left untreated (first row), treated with 50 μM Gemfibrozil (GFZ, second row), treated with 5 μM methyl-β-cyclodextrin and 50 μM GFZ (third row), or with 10 μM 25-hydroxycholesterol (25-HC, bottom panel). Free cholesterol in plasma membranes was visualized by Filipin III staining (0.05 mg/ml), and DNA was visualized by Propidium iodide (PI) staining (5 μg/ml). Images representative of three independent experiments. Scale bar indicated next to images.

Supplemental Figure 6:

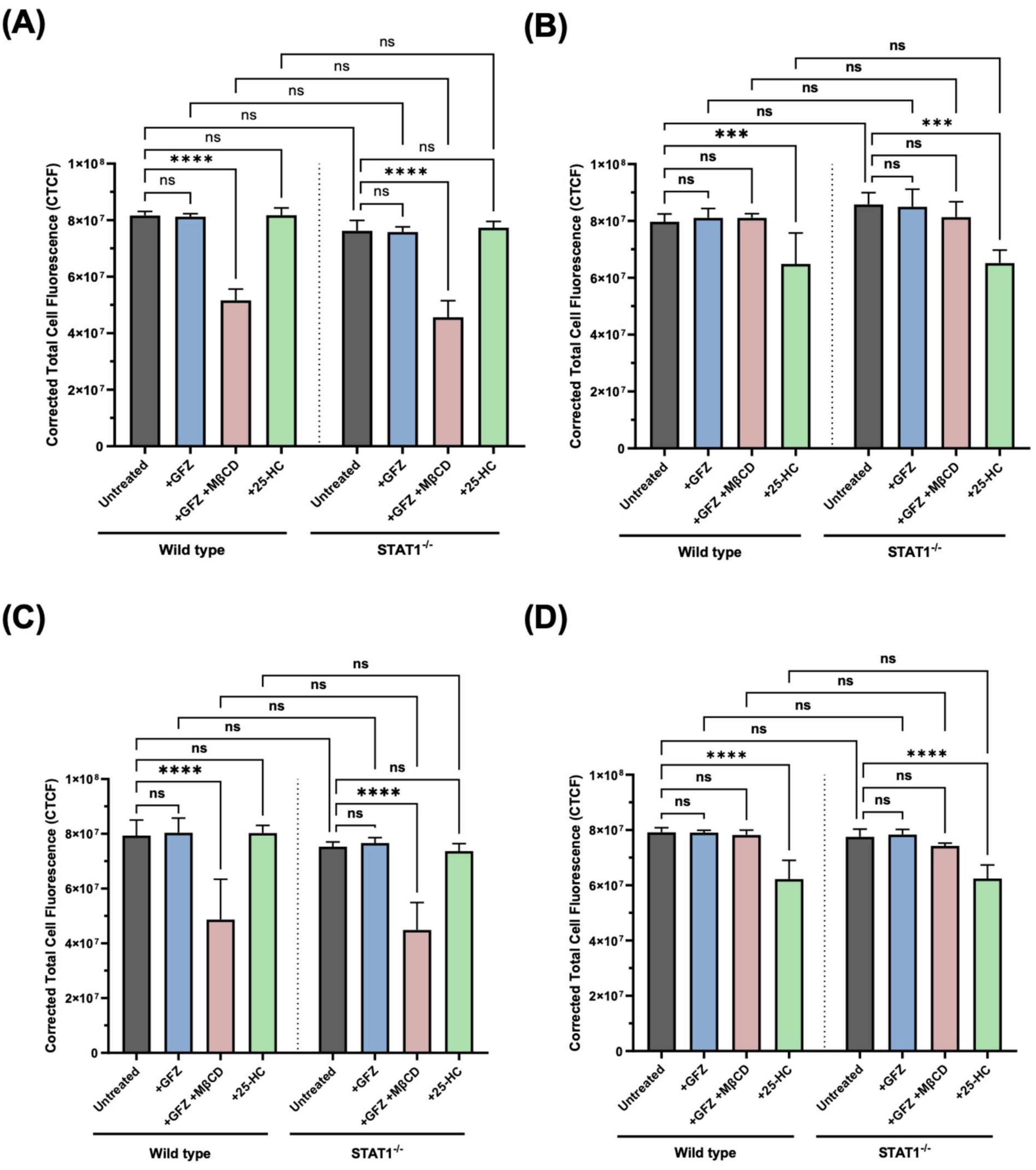

Supplemental Figure 6: Quantification of Filipin III intensity. Quantification of total free cholesterol by Filipin III signal intensity in wild-type and STAT1$^{-/-}$ HEp-2 cells, which were uninfected (grey), treated with 50 µM Gemfibrozil (GFZ, blue), treated with 5 µM methyl-β-cyclodextrin (MβCD) and 50 µM GFZ (red), or with 10 µM 25-hydroxycholesterol (25-HC, green). Graphs show signal intensity of rRSV-A-0594-eGFP-infected wild-type and STAT1$^{-/-}$ HEp-2 cells at 0 hpi **(A)** and 48 hpi **(B)** or of uninfected wild-type and STAT1$^{-/-}$ HEp-2 cells at 0 hpi **(C)** and 48 hpi **(D)**. Statistical analysis: one-way ANOVA with Tukey‘s post-hoc test. Mean ± SD of three independent experiments is shown.

Supplemental Figure 7:

(A)

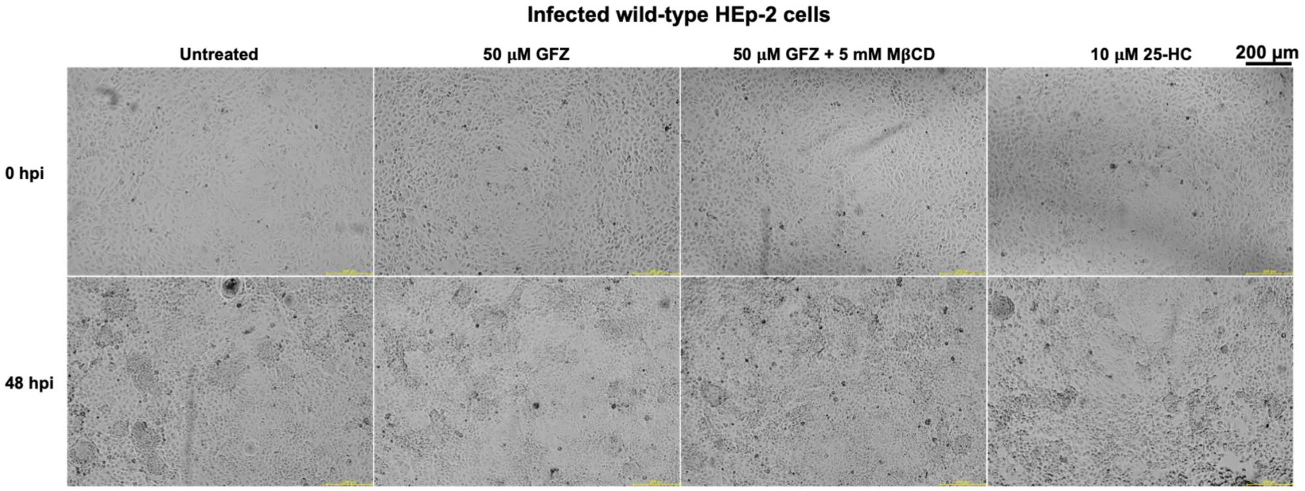

(B)

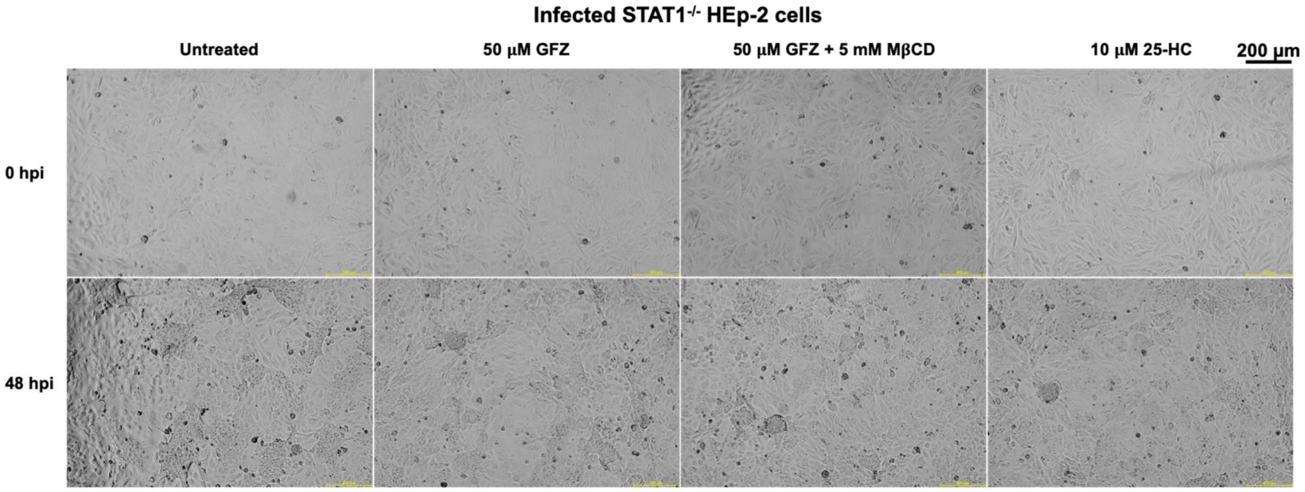

Supplemental Figure 7: Bright-field images of infected wild-type and STAT1$^{-/-}$ HEp-2 cells. Representative bright-field images of rRSV-A-0594-eGFP-infected wild-type **(A)** and STAT1$^{-/-}$ **(B)** HEp-2 cells (MOI 0.05) at 48 hpi. Images correspond to fluorescence images in Figure 4 (Filipin III staining). Cells were left untreated (first row), treated with 50 µM Gemfibrozil (GFZ, second row), treated with 5 µM methyl-β-cyclodextrin and 50 µM GFZ (third row), or with 10 µM 25-hydroxycholesterol (25-HC, bottom panel). Images representative of three independent experiments. Scale bar indicated next to images.

Supplemental Figure 8:

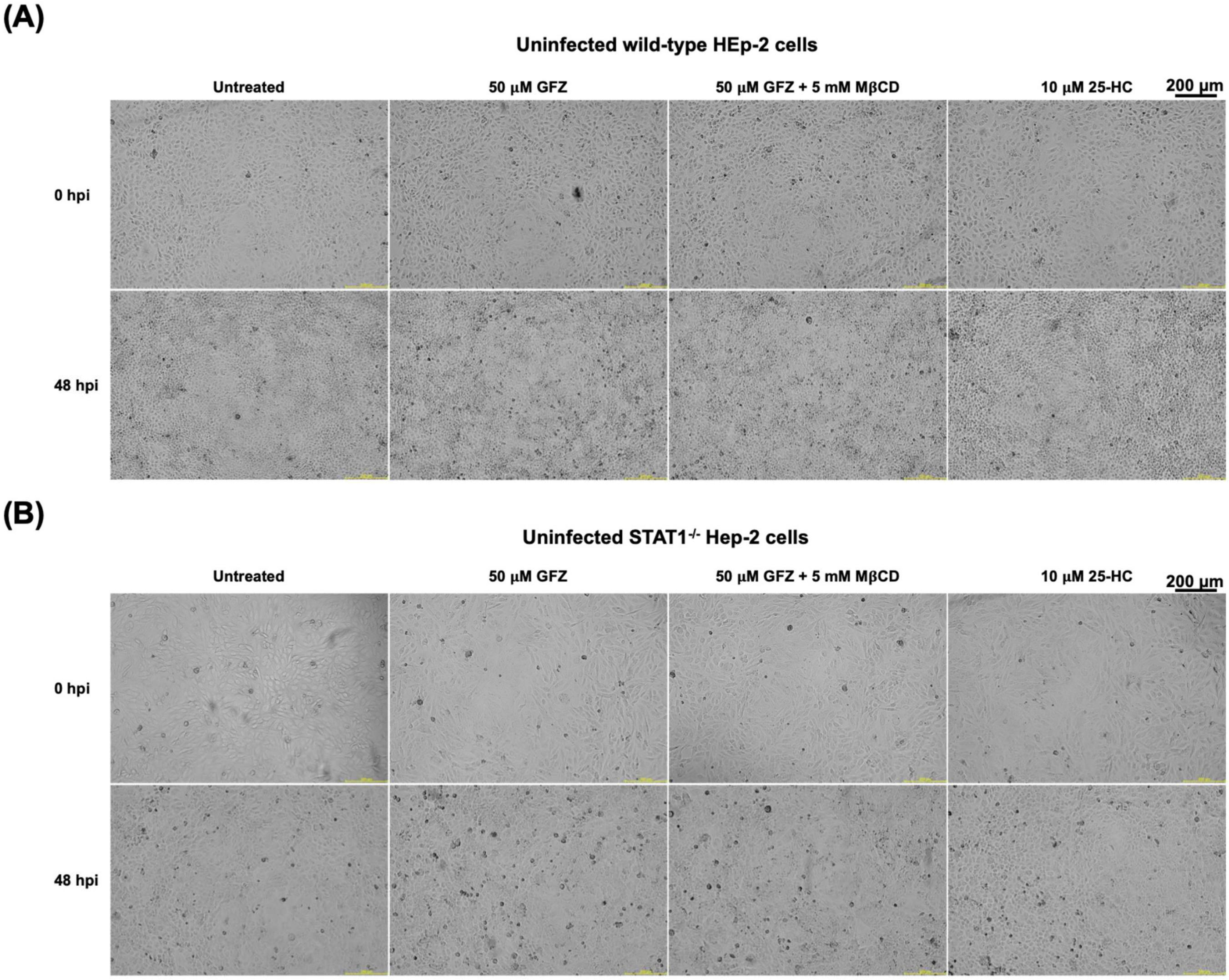

Supplemental Figure 8: Bright-field images of uninfected wild-type and STAT1$^{-/-}$ HEp-2 cells. Representative bright-field images of uninfected wild-type **(A)** and STAT1$^{-/-}$ **(B)** HEp-2 cells (MOI 0.05) at 48 hpi. Images serve as a control for Figure 4 (Filipin III staining) and correspond to fluorescence images shown in Supplemental Figures 4 and 5. Cells were left untreated (first row), treated with 50 µM Gemfibrozil (GFZ, second row), treated with 5 µM methyl-β-cyclodextrin and 50 µM GFZ (third row), or with 10 µM 25-hydroxycholesterol (25-HC, bottom panel). Images representative of three independent experiments. Scale bar indicated next to images.

Supplemental Figure 9:

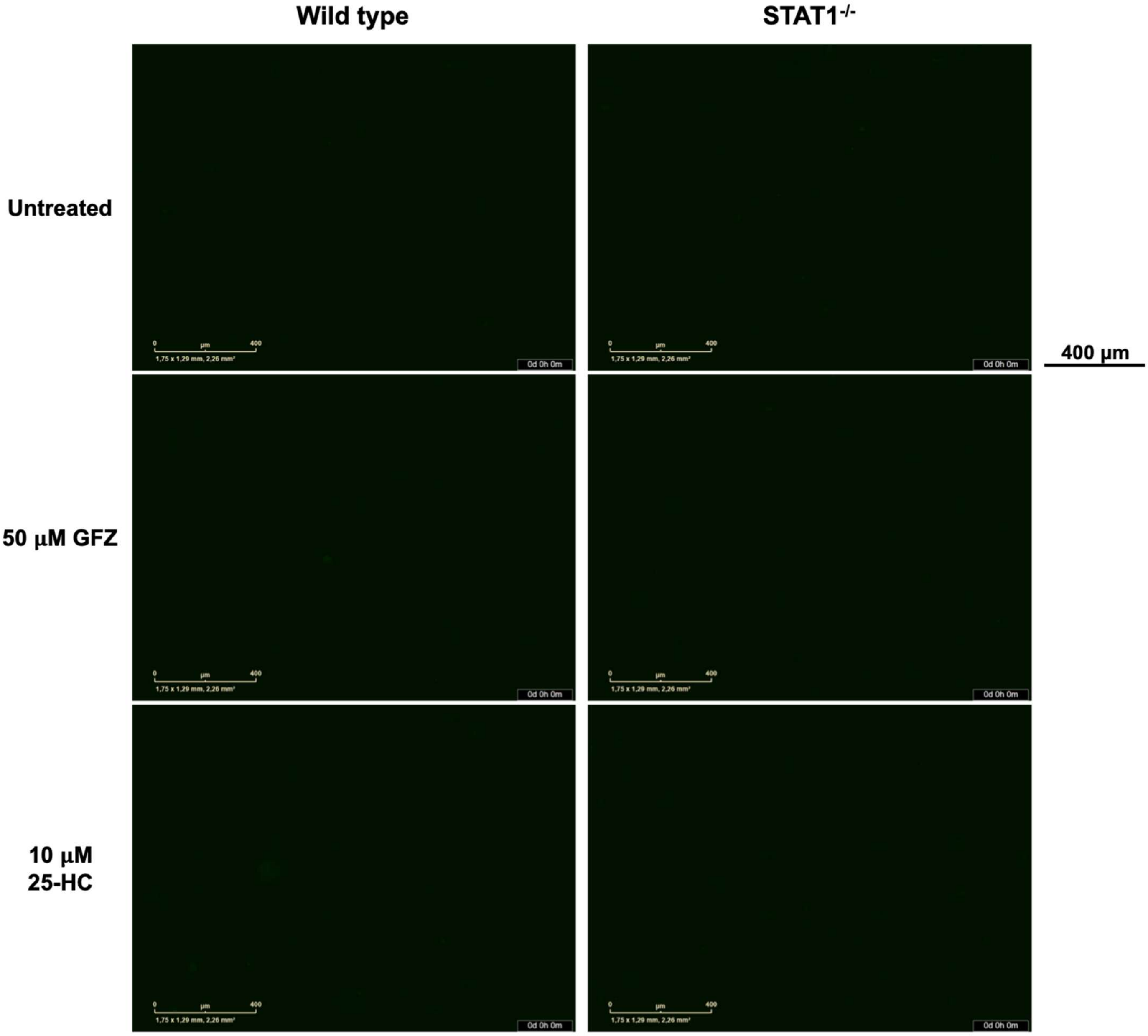

Supplemental Figure 9: Incucyte Live-cell imaging of infected wild-type and STAT1-/- HEp-2. Representative videos from IncuCyte live-cell imaging of rRSV-A-0594-eGFP-infected wild-type (left) and STAT1$^{-/-}$ (right) HEp-2 cells (MOI 0.05), which were left untreated, treated with 50 µM Gemfibrozil (GFZ), or with 10 µM 25-hydroxycholesterol (25-HC) until 48 hpi. Videos of the GFP channel are shown. Videos representative of three independent experiments. Scale bar indicated next to videos.

Supplemental Figure 10:

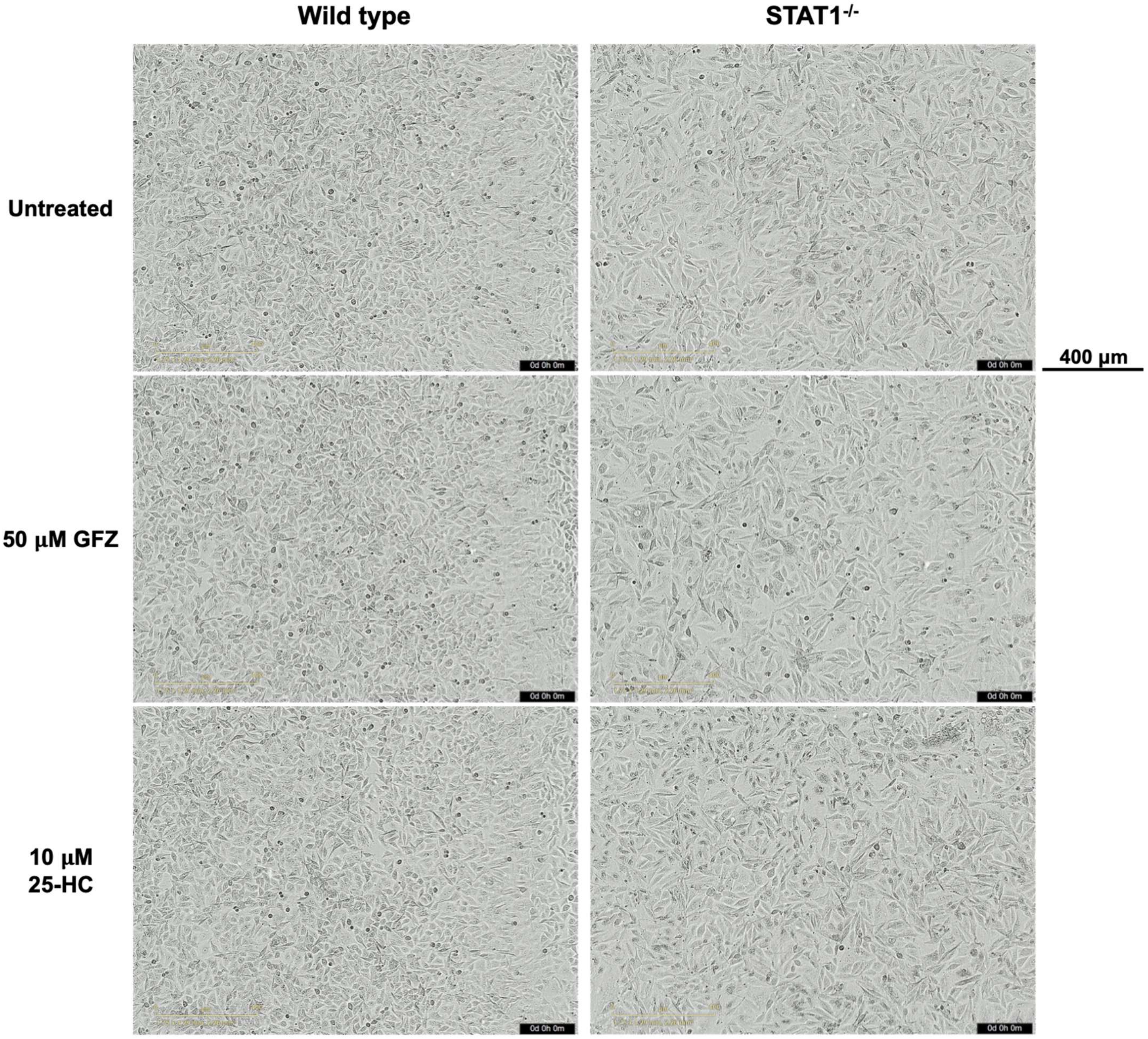

Supplemental Figure 10: Bright-field IncuCyte Live-cell imaging of infected wild-type and STAT1$^{-/-}$ HEp-2. Representative videos from IncuCyte live-cell imaging of rRSV-A-0594-eGFP-infected wild-type (left) and STAT1$^{-/-}$ (right) HEp-2 cells (MOI 0.05), which were left untreated, treated with 50 µM Gemfibrozil (GFZ), or with 10 µM 25-hydroxycholesterol (25-HC) until 48 hpi. Phase-contrast videos are shown and correspond to videos from Supplemental Figure 9. Videos representative of three independent experiments. Scale bar indicated next to videos.

Supplemental Figure 11:

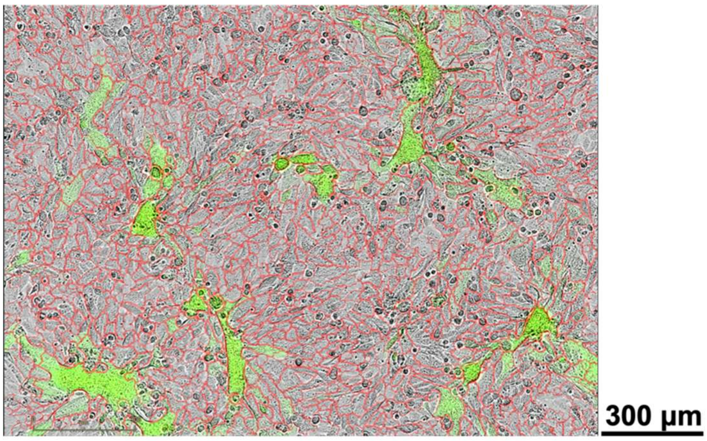

Supplemental Figure 11: Overlay and segmentation of infected HEp-2 cells. Representative bright-field image overlayed with eGFP signal of rRSV-A-0594-eGFP-infected, untreated STAT1$^{-/-}$ HEp-2 cells (MOI 0.05) at 48 hpi. The overlay image corresponds to the fluorescence image shown in Figure 5a (STAT1$^{-/-}$ HEp-2 cells, infected, untreated, 48 hpi), illustrating how the cell borders were set to distinguish infected, individual cells in proximity from actual syncytia. A detailed description of the settings is provided in the Materials and Methods section of the manuscript. Images representative of three independent experiments (three technical replicates). Scale bar indicated next to image.

Supplemental Figure 12:

(A)

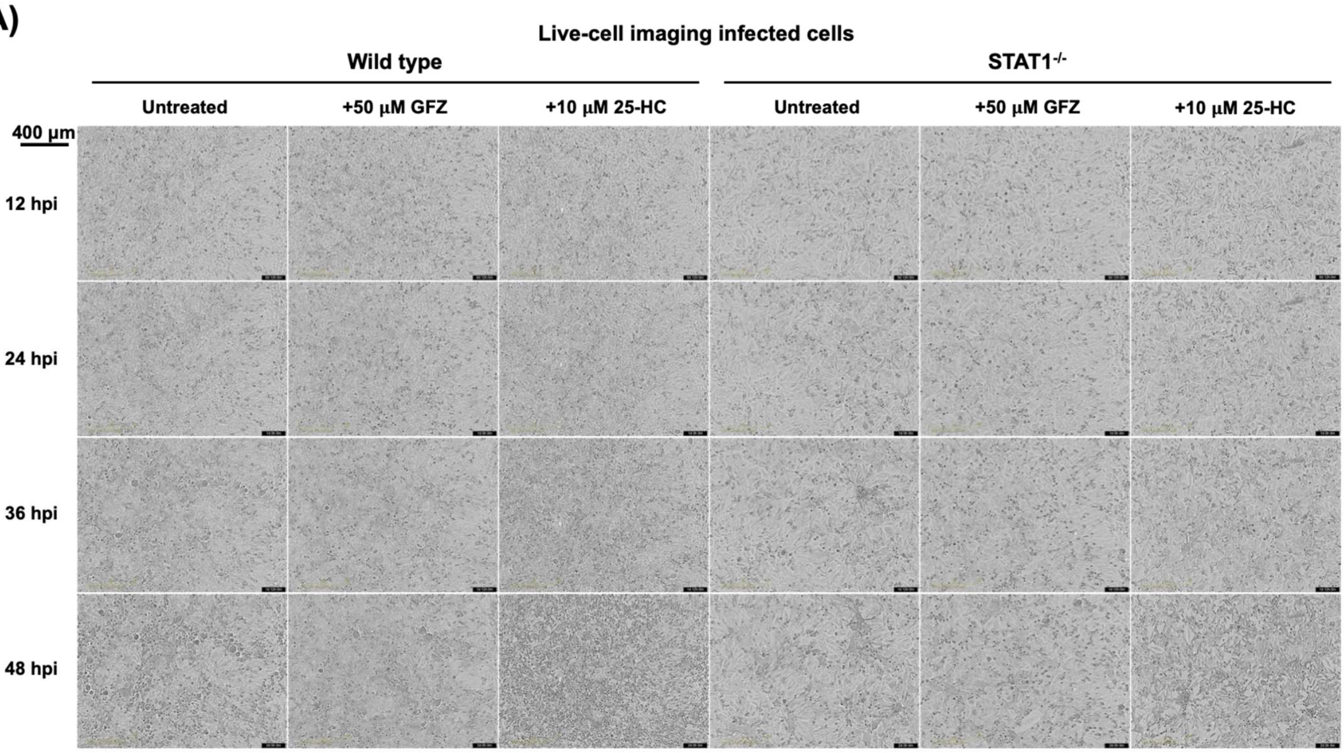

(B)

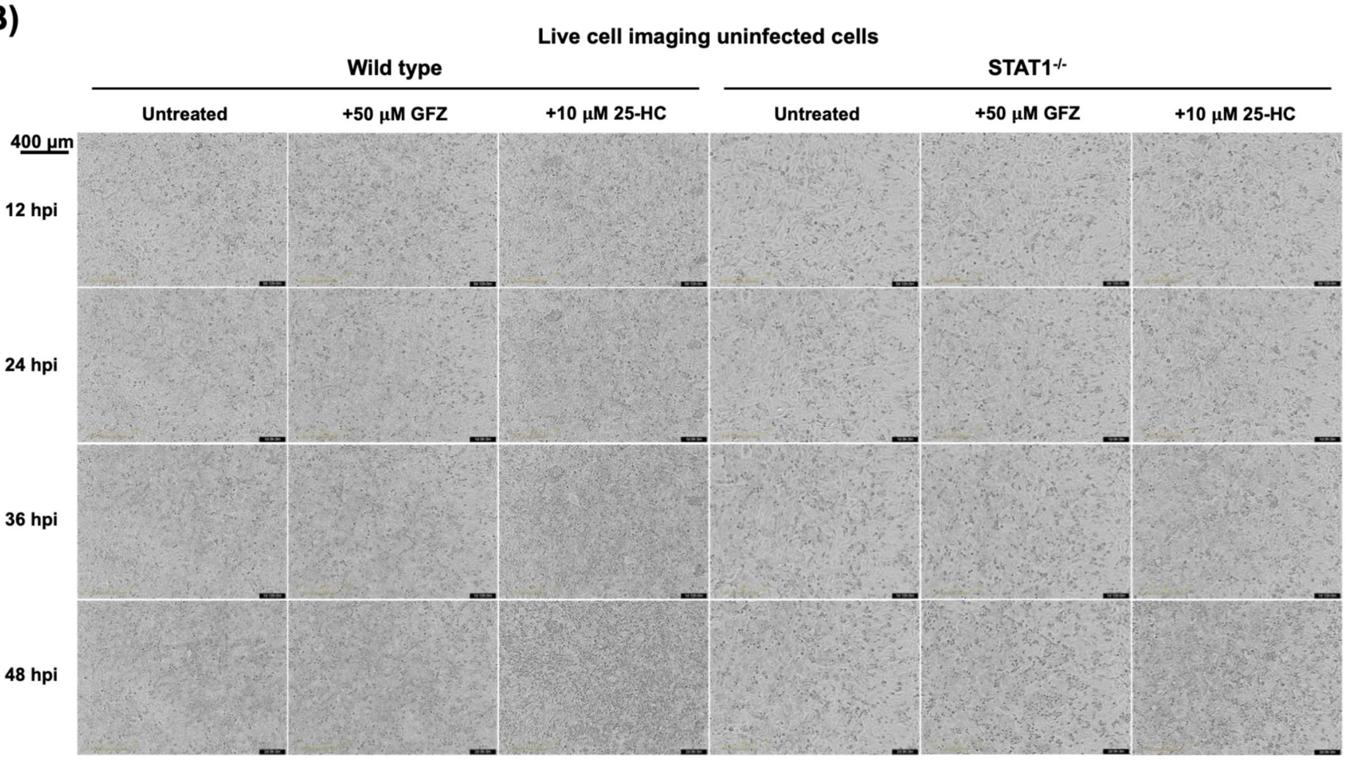

Supplemental Figure 12: Bright-field images of IncuCyte Live-Cell imaging. Representative bright-field images of rRSV-A-0594-eGFP-infected **(A)** or uninfected **(B)** wild-type (left) and STAT1$^{-/-}$ (right) HEp-2 cells (MOI 0.05), which were left untreated (left column), treated with 50 µM Gemfibrozil (GFZ, middle column), or with 10 µM 25-hydroxycholesterol (25-HC, right column) at 12, 24, 36, and 48 hpi.

Bright-field images in **(A)** correspond to fluorescence images shown in Figure 5a, while images in **(B)** represent uninfected controls for Figure 5a. Images representative of three independent experiments. Scale bar indicated next to images.

Supplemental Figure 13:

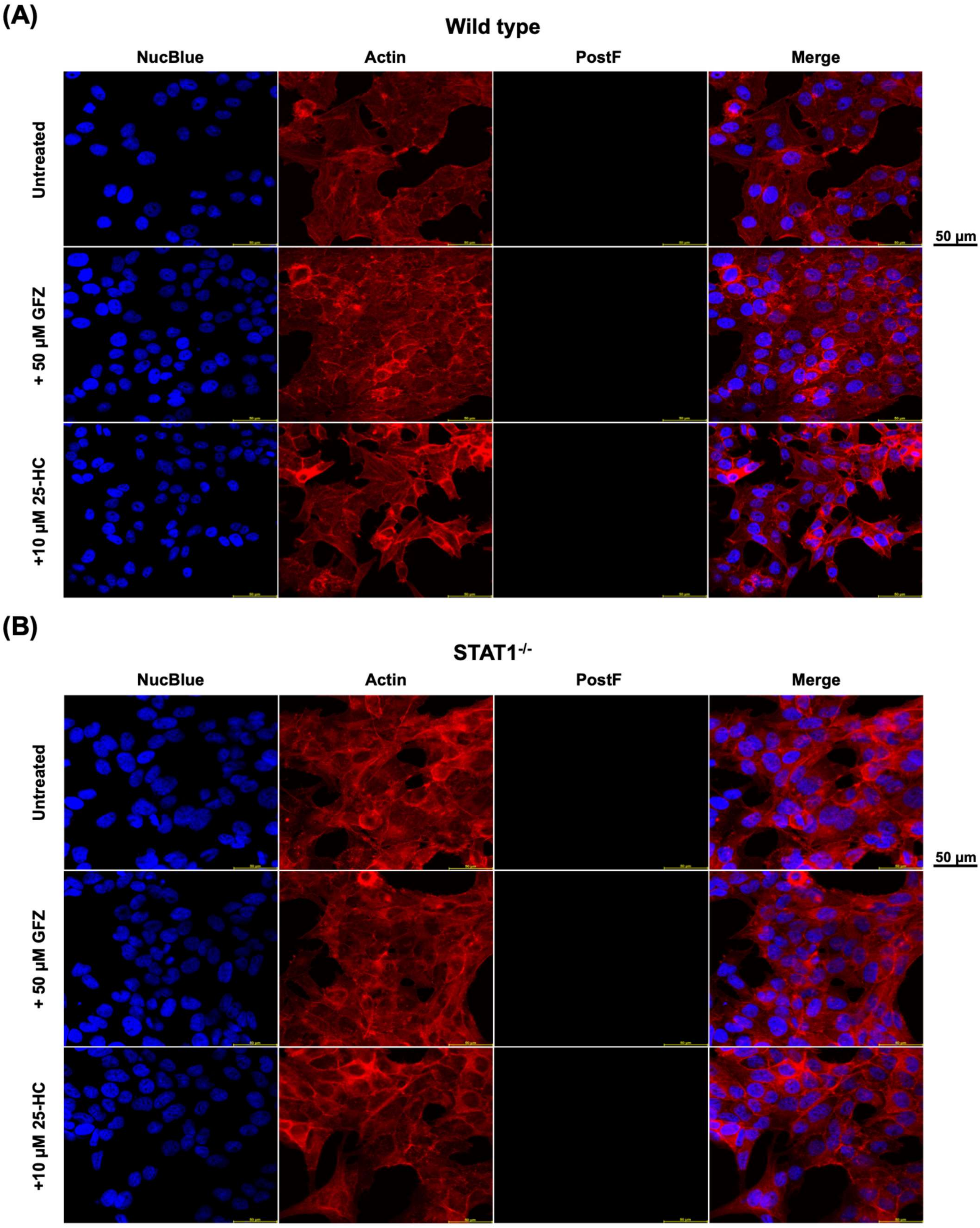

Supplemental Figure 13: Confocal images of uninfected wild-type and STAT1$^{-/-}$ HEp-2 cells. Confocal microscopy images of uninfected wild-type **(A)** and STAT1$^{-/-}$ **(B)** HEp-2 cells (MOI 0.05) at 48 hpi. Infected cells were left untreated (top), treated with 50 µM Gemfibrozil (GFZ, middle), or 10 µM 25-hydroxycholesterol (25-HC, bottom). Cells were stained for RSV postfusion F protein, actin (ActinRed555), and nuclei (NucBlue). Images represent controls for infected cells in Figure 6. Images representative of three independent experiments. Scale bar indicated next to images.

Supplemental Table 1: Gene start and end positions for RSV transcripts

Transcript	Gene start position	Gene end position
NS1	99	518
NS2	628	1002
N	1140	2315
P	2347	3072
M	3255	4025
SH	4295	4489
G	4681	5646
F	5726	7450
M2.1	7669	8253
M2.2	8228	8494
L	8561	15058

Supplemental Table 2: Expression trends of Gene clusters 1-4 in mock-infected samples

Gene Cluster 1: Genes directly involved in cholesterol biosynthesis

Gene name	Expression trend	p value
HMGCS1	↓	2.30E-41
MSMO1	↓	1.40E-40
FDFT1	↓	1.30E-36
HMGCR	↑	1.50E-36
NSDHL	↓	5.50E-34
SQLE	↓	1.00E-32
MVK	↓	4.40E-30
FDPS	↓	1.10E-28
MVD	↓	3.20E-26
CYP51A1	↓	4.50E-26
TM7SF2	↓	2.20E-25
EBP	↓	3.60E-20
HSD17B7	↓	3.40E-18
DHCR24	↓	1.90E-16
DHCR7	↑	2.40E-13
SC5D	↑	1.70E-10
LSS	↓	6.80E-10
GGPS1	↓	7.20E-08

Gene Cluster 2: Transcription (co-)factors controlling cholesterol biosynthesis

Gene name	Expression trend	p value
TBL1X	↓	1.10E-18
MTF1	↓	3.50E-14
NFYB	↑	5.80E-13
CARM1	↓	4.90E-11
NCOA6	↓	1.10E-08
CREBBP	↓	2.30E-08
SREBF2	↓	3.20E-07
NFYC	↓	2.00E-06

Gene Cluster 3: Indirect modulators of cholesterol homeostasis

Gene name	Expression trend	p value
SCD	↓	3.10E-23
ACAA2	↓	6.90E-19
ELOVL6	↓	5.70E-10
GPAM	↓	1.20E-06
FASN	↓	4.90E-06
ACACA	↓	1.30E-04

Gene Cluster 4: Direct regulators of cholesterol biosynthesis and homeostasis

Gene name	Expression trend	p value
INSIG1	↓	4.80E-66
ACAT2	↓	3.70E-25
SEC24C	↓	2.20E-21
PRKAA2	↓	5.10E-15
KPNB1	↓	6.40E-13
INSIG2	↑	4.20E-10

Supplemental Table 3: Gene clusters involved in inflammatory response and

Gene Cluster 1: Innate immune sensors				
**Gene name**	**Expression trend compared to Mock: Wild Type**	**p value**	**Expression trend compared to Mock: STAT1-/-**	**p value**
IFIH1	↑	2.00E-17	↑	0.001
DDX58	↑	3.00E-15	↑	0.005
IFI16	↑	1.70E-05	↑	0.062

Gene Cluster 2: Antigen processing and presentation				
**Gene name**	**Expression trend compared to Mock: Wild Type**	**p value**	**Expression trend compared to Mock: STAT1-/-**	**p value**
HLA-B	↑	6.20E-07	↑	16,669
TAP1	↑	4.00E-08	↑	0.408
PSMB8	↑	8.20E-08	↓	2.40E-04
B2M	↑	4.60E-10	↓	8.30E-25
HLA-C	↑	0.041	↑	0.414

Gene Cluster 3: Host factors involved in viral replication				
**Gene name**	**Expression trend compared to Mock: Wild Type**	**p value**	**Expression trend compared to Mock: STAT1-/-**	**p value**
EIF4G1	↓	7.50E-13	↑	1.50E-23
NUP98	↓	4.60E-04	↑	0.345
DDX3X	↓	0.005	↑	0.797
NXF1	↑	5.00E-05	↑	0.078
RANBP2	↓	0.002	↑	0.205

Gene Cluster 4: Stress response regulators				
**Gene name**	**Expression trend compared to Mock: Wild Type**	**p value**	**Expression trend compared to Mock: STAT1-/-**	**p value**
BAX	↓	0.014	↓	0.388
BNIP3	↑	1.80E-09	↑	8.10E-68
CFLAR	↑	0.003	↑	0.032
HSPA8	↑	0.00	↑	0.022
CASP1	↑	6.80E-14	↑	2.80E-19

Gene Cluster 5: Immune signaling and transcription regulators				
**Gene name**	**Expression trend compared to Mock: Wild Type**	**p value**	**Expression trend compared to Mock: STAT1-/-**	**p value**
STAT1	↑	5.70E-06	↑	0.494
STAT3	↑	5.90E-06	↑	0.047
JAK1	↑	1.40E-05	↑	0.386
IRF7	↑	3.40E-13	↑	0.022
IRAK1	↑	6.50E-07	↑	0.002

Gene Cluster 6: Interferon-stimulated genes				
**Gene name**	**Expression trend compared to Mock: Wild Type**	**p value**	**Expression trend compared to Mock: STAT1-/-**	**p value**
ISG15	↑	3.30E-24	↑	3.20E-12
IFIT1	↑	3.00E-33	↑	4.10E-10
RSAD2	↑	2.00E-07	↑	4.00E-05
TRIM25	↑	0.0222	↑	0.384
OAS1	↑	3.30E-09	↑	0.024

Supplemental Table 4: Gene clusters linked to cholesterol biosynthesis in infected samples

B: Enriched pathways linked to cholesterol biosynthesis

Gene Cluster 1: Genes directly involved in cholesterol biosynthesis

Gene name	Expression trend compared to Mock: Wild Type	p value	Expression trend compared to Mock: STAT1-/-	p value
FDPS	↓	6.50E-85	↑	1.70E-61
MSMO1	↓	4.40E-116	↑	6.90E-38
DHCR7	↓	3.20E-41	↑	2.90E-29
SQLE	↓	4.90E-29	↑	1.50E-28
MVD	↓	3.30E-42	↑	1.90E-23
HMGCR	↓	4.40E-10	↑	1.80E-22
HSD17B7	↓	7.40E-59	↑	3.20E-22
FDFT1	↓	4.50E-56	↓	5.80E-21
HMGCS1	↓	1.80E-15	↑	4.70E-12
LSS	↓	9.10E-07	↓	7.60E-08
DHCR24	↓	1.00E-06	↑	2.80E-10
EBP	↓	2.50E-36	↑	5.80E-10
MVK	↓	8.10E-18	↑	1.50E-09
CYP51A1	↓	6.10E-27	↑	2.80E-09
NSDHL	↓	3.10E-17	↑	1.30E-08
TM7SF2	↓	4.90E-18	↓	3.50E-08
GGPS1	↓	4.70E-07	↑	9.70E-06
SC5D	↓	6.50E-15	↓	6.90E-08
IDI1	↓	1.00E-31	↓	3.50E-15

Gene Cluster 2: Transcription (co-)factors controlling cholesterol biosynthesis

Gene name	Expression trend compared to Mock: Wild Type	p value	Expression trend compared to Mock: STAT1-/-	p value
CARM1	↓	6.10E-07	↑	3.90E-07
SCAP	↓	0.018	↑	2.00E-04
HELZ2	↑	4.10E-14	↑	0.005
MTF1	↑	1.30E-05	↑	0.359
CREBBP	↓	1.60E-07	↑	0.023
TBL1X	↓	1.30E-06	↑	0.295
NCOA6	↓	3.50E-06	↑	0.078
SREBF2	↓	9.00E-06	↑	2.40E-08

Gene Cluster 3: Indirect modulators of cholesterol homeostasis

Gene name	Expression trend compared to Mock: Wild Type	p value	Expression trend compared to Mock: STAT1-/-	p value
ACLY	↓	6.60E-04	↓	5.80E-12
CYB5R3	↓	0.449	↑	1.30E-05
FASN	↓	4.70E-07	↑	1.80E-27
ELOVL6	↓	2.30E-05	↑	6.50E-12
ACACA	↓	0.043	↑	8.90E-04
SCD	↓	3.80E-08	↓	2.70E-09
G6PD	↓	7.50E-08	↑	7.50E-05

Gene Cluster 4: Direct regulators of cholesterol biosynthesis and homeostasis

Gene name	Expression trend compared to Mock: Wild Type	p value	Expression trend compared to Mock: STAT1-/-	p value
INSIG1	↓	3.40E-30	↑	3.3E.34
INSIG2	↓	2.50E-06	↑	1.40E-16
ACAT2	↓	7.70E-54	↑	4.10E-26
